# Density Functional Theory Calculations: A Useful Tool to Investigate Mechanisms of 1,3-Dipolar Cycloaddition Reactions

**DOI:** 10.3390/ijms25021298

**Published:** 2024-01-20

**Authors:** Maria Assunta Chiacchio, Laura Legnani

**Affiliations:** 1 Department of Drug and Health Sciences, University of Catania, Viale A. Doria 6, 95125 Catania, Italy; 2 Department of Biotechnology and Biosciences, University of Milano-Bicocca, Piazza della Scienza 2, 20126 Milano, Italy

**Keywords:** DFT calculations, dipolar cycloadditions, aza-ylides, nitrile oxides, azomethine ylides, nitrones

## Abstract

The present review contains a representative sampling of mechanistic studies, which have appeared in the literature in the last 5 years, on 1,3-dipolar cycloaddition reactions, using DFT calculations. Attention is focused on the mechanistic insights into 1,3-dipoles of propargyl/allenyl type and allyl type such as aza-ylides, nitrile oxides and azomethyne ylides and nitrones, respectively. The important role played by various metal–chiral–ligand complexes and the use of chiral eductors in promoting the site-, regio-, diastereo- and enatioselectivity of the reaction are also outlined.

## 1. Introduction

### 1.1. Computational Methods

In recent years, theoretical and computational studies have increasingly accompanied experimental data, providing support in the conformational analysis of molecules, biomolecules and their bioactive synthetic analogues and in the explanation of the mechanisms of organic reactions.

The conformational analysis of organic molecules is based on the principle that molecules are not rigid and their kinetic energy at room temperature is large enough to let all atoms exhibit permanent molecular movement. This fact means that the absolute positions of atoms in a molecule and of the molecule as a whole are not fixed and that the relative location of substituents on a single bond is different at each moment. So, every molecule, with one or more single bonds, exists in many different rotamers or conformers. Low-energy conformers make the major contribution to the overall population. A transformation from one geometry to another is primarily related to changes in the torsional angles of single bonds. A complete overview of the conformational potential surface of molecules can be gained by theoretical techniques, and numerous methods for conformational analysis, which are able to identify all minima on the potential energy surface, have been developed. The number of minima increases with the number of rotatable bonds. Thus, the detection of all minima becomes difficult and time consuming. The most used techniques for conformational analyses are systematic search procedures, performed by systematically varying each of the torsion angles to generate all possible conformations, and molecular dynamics, which reproduces the time-dependent motional behavior of a molecule [1]. 

Another very important application of theoretical studies, which has found widespread application in recent decades, is the clarification of various mechanisms of organic reactions, locating reactants, intermediates, transition states and products along the reaction pathways.

In order to locate the different geometries of the molecules or the different points along a reaction coordinate, different methods can be used. In computational chemistry, quantum mechanical methods are very important tools. 

They are based on the calculation of the interaction energies between a nucleus–nucleus, electron–nucleus and electron–electron for all atoms of the studied molecules and on the solution of the corresponding Schrödinger Equation (1).

HΨ = EΨ(1)

Quantum mechanical methods allow for calculating the energy and electronic structure of molecular systems in different spatial configurations, to analyze the breaking and formation of bonds and to study the chemical reactivity. Nevertheless, they have a disadvantage in their high computational cost. 

Nowadays, a quantum mechanical approach that can locate widespread diffusion, is density functional theory (DFT) [1], which is based on the resolution of the Kohn–Sham equations. Calculation times are shorter with respect to other quantum mechanical methods, but the accuracy of the results is preserved. 

DFT calculations, on the basis of the Hohenberg–Kohn theorem [2], consider the electronic energy of the molecular ground state to be completely determined by electron density (ρ). In fact, the wave function, depending on one spin and three spatial coordinates for every electron of the studied systems, is not intuitive for compounds with more than one electron. Therefore, an important target is the possibility of finding a physical observable that permits the *a priori* construction of the Hamiltonian operator. The dependence of the Hamiltonian on the positions and atomic numbers of nuclei and on the total numbers of electrons suggests ρ as a useful research observable since, when integrated over the entire space, it provides the total number of electrons.

The correspondence between the electron density (ρ) of a system and its energy represented the basis of DFT theory and it is of crucial importance considering a comparison with the wave function.

In fact, while the complexity of the wave function increases with the number of electrons, ρ, which only depends on the three spatial coordinates, it presents the same number of variables independently from the system’s dimensions. On the basis of this approach, the electron density, which determines the external potential, furnishes the Hamiltonian, from which the wave function can be obtained. Finally, once these parameters are determined, the energy of the system can be computed. 

However, this direction does not award any simplification over the Molecular Orbital (MO) theory, since the final step still requires solving the Schrödinger equation, which is very difficult because of the electron–electron interaction term of the Hamiltonian. In 1965, Kohn and Sham [3] realized that it was possible to solve the problem by considering a non-interacting system of electrons and evaluating the kinetic energy, T. Because of this approximation, the calculated T is lightly undervalued, but the difference with respect to the real value of the energy is small and is included in the Exchange-Correlation term (E_xc_). So, in general, the DFT energy results in

E_DFT_ = T(ρ) + E_ne_(ρ) + J(ρ) + E_xc_(ρ)

T(ρ) is the kinetic energy in the hypothesis that electrons do not interact each other;

E_ne_(ρ) is nucleus–electron attraction energy;

J is Coulomb energy between electrons;

E_xc_ is, as said above, exchange and correlation term.

Between the different DFT methods, the B3LYP [4] hybrid functional (a hybrid method obtained by combining the five functionals Becke, Slater, HF exchange, LYP and VWN5 correlation) gave the most accurate results for a large number of compounds and in particular for organic molecules, finding wide application by computational organic chemists.

In the last few years, a significant number of new density functionals, for applications of Kohn–Sham density functional theory, in chemistry and physics were developed. For example, the Thrular’s hybrid metafunctionals M06 and M06-2X are widely used for their broad applicability, in particular in transition metal chemistry and in the study of excited-states.

Moreover, it is worthwhile pointing out that during the calculations, molecules can be considered in the gas phase and so treated as isolated, non-interacting species with enormous facilities for theoretical modeling. However, this approach is not accurate enough for water-soluble biomolecules. In fact, the solvent phase deeply influences the results because of the hydrogen bond interactions and the charge polarization differences from the gas and the water phase. When the solvent is considered, the first point is the number of solvent molecules to add to the system. To overcome this problem, the so-called implicit models or continuum solvation models have been developed [1], in which the solvent is replaced by a continuous electric field. One family of these methods is called the Self-Consistent Reaction Field (SCRF) that considered the solvent as a continuum of uniform dielectric constant (ε). Perhaps the most popular SCRF method is the Tomasi’s Polarized Continuum Model (PCM) [5], from which also a conductor-like modification was described: the C-PCM method [6]. 

Despite the accuracy of QM methods, they cannot be used to study some biochemical systems, such as enzymes, because of their dimensions. Nevertheless, the use of methods that are less time-demanding (i.e., MM methods) to investigate the breaking and forming of bonds during a reaction is not feasible. To overcome these limitations, hybrid quantum mechanics/molecular mechanics (QM/MM) simulations were developed. These approaches consist of the treatment of the region, in which the chemical process takes place, with a QM method, while the larger part of the system is modeled by a molecular mechanics force field [7].

In the last five years, DFT calculations were applied by organic chemists to support hypotheses on different reactions mechanisms. A very interesting field of application was the investigation of the synchronous or asynchronous technique of bonds formation in concerted mechanisms with particular regard to the 1,3-dipolar cycloadditions (1,3-DC) [8]. A very significant support to the rationalization of the various reaction pathways of pericyclic reactions is offered by the Frontiers Molecular Orbital (FMO) theory, which is based on the idea that a good approximation of reactivity can be obtained not considering all the orbitals of the system but rather only the highest occupied orbital of one reactant (HOMO) and the lowest unfilled (lowest unoccupied orbital, LUMO) of the other one.

Another very useful tool to interpret results and rationalize the mechanism of pericyclic reactions is the Houk/Bickelhaupt activation strain model [9]. With this approach, activation barriers, that determine reaction rates, can be obtained. In detail, in the case of bimolecular processes, the sum of the energies needed to distort the addends to reach the same geometry as in the TSs, and the interaction energies between the two distorted reactants, furnish the activation energies. The changes of these values during the reaction clarify the factors that control the reactivity.

In many of the reviewed works in the following sections, these important principles of physical organic chemistry are used to interpret results.

### 1.2. 1,3-Dipolar Cycloaddition

The 1,3-dipolar cycloadditions reactions can be considered the most productive heterocyclic synthesis that allow, using a single step, the introduction of three or four stereogenic centers in a stereospecific manner. The addends of this type of reactions are dipolarophiles, i.e., compounds with π bonds and 1,3-dipoles that can be distinguished in linear propargyl/allenyl types or allyl types.

The concerted cycloaddition shows a regioselectivity and stereoselectivity that can be appropriately rationalized with the frontier molecular orbital theory, studying the interactions between the LUMO and HOMO of dipole and dipolarophile in the *endo* and *exo* transition states. Moreover, the regio, diastereo and enantioselectivity of the process can also depend on the presence of metals in the reaction environment. In fact, metal ions, acting as Lewis’s acids, can alter not only the orbital coefficients of the reacting atoms but also the energy of the frontier orbitals of the reacting species [10]. 

All of the information reported above highlights the importance of computational studies aimed to predict or clarify the mechanistic and stereochemical outcomes of these reactions.

With the development of computational methodologies, in the last few decades, the growing interest of organic chemists for their application in the study of 1,3-dipolar cycloaddition reactions has been demonstrated by the increasing number of publications in this area [11,12,13,14,15,16].

In this review, we are going to show the recent applications (2018–2023) of DFT methods to mechanistic speculation on reaction routes of 1,3-CDs classified on the basis of the different involved dipoles, i.e., 1,3-dipoles of propargyl/allenyl type (aza-ylides and nitrile oxides) and allyl type (azomethine ylides and nitrones) that appeared in the literature in the last few years.

## 2. Computational Mechanistic Studies of 1,3-Dipoles

### 2.1. 1,3-Dipoles of Propargyl-Allenyl Type

Dipoles of the propargyl-allenyl type present a linear structure in which a triple bond is present in canonical form.

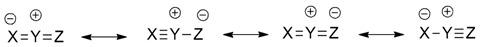



The main dipoles belonging to this category are: aza-ylides (I), diazoalkanes (II), nitrile oxides (III), and nitrilimines (IV).

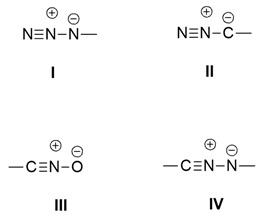



In the following sections, only I and III will be considered.

#### 2.1.1. Aza-Ylides

Aza-ylides represent a very important tool for the synthesis of complex heterocyclic compounds and are involved as special frameworks in the so-called “click chemistry”. When in 2001 [17], Sharpless described the ideal conditions for click reactions, also the 1,3-dipolar cycloadditions were listed as candidates for application of this new branch.

In 2002, the first copper-catalyzed azide–alkyne cycloadditions were reported [18]. They were conducted in mild reaction conditions, with high yields, and the formation of only one regioisomer (i.e., 1,4-disubstituted 1,2,3-triazole derivatives). In this way, organic azides gained increased attention in this synthetic field and obtained an enormous success as 1,3-dipoles for the preparation of compounds with applications in life sciences.

**Figure 1:**
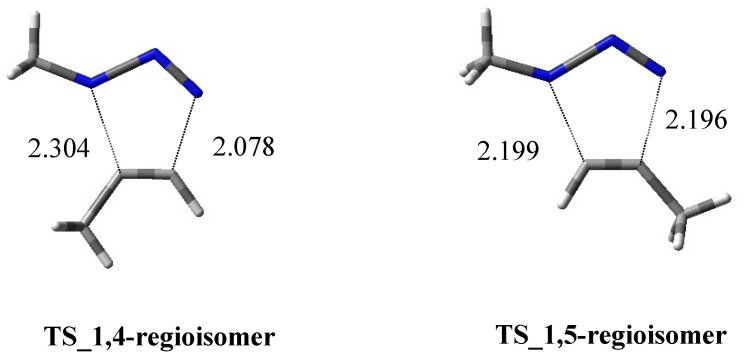
Three-dimensional (3D) plots of the TSs of the uncatalyzed route leading to 1,4- and 1,5-regioisomer, respectively.

In 2018, Ben El Ayouchia [19] investigated and explained the uncatalyzed and copper(I)-catalyzed [3 + 2] cycloaddition of methyl azide with propyne using DFT calculations at the B3LYP/6-31G(d) level. In the case of the uncatalyzed route, two regioisomeric paths (1,4- and 1,5-approaches) were studied, showing similar high activation energies of 18.84 (**TS1**) and 18.51 (**TS2**) kcal/mol, respectively (Figure 1). These values give an explanation to the lack of regioselectivity of the coupling conducted in the absence of catalysts. The asynchronicity was evaluated and shown to be higher for the 1,5-regioisomer process with respect to the 1,4-one. In general, the formation of triazoles is highly exothermic, and calculations showed an asynchronous one-step mechanism with a very non-polar character.

**Figure 2:**
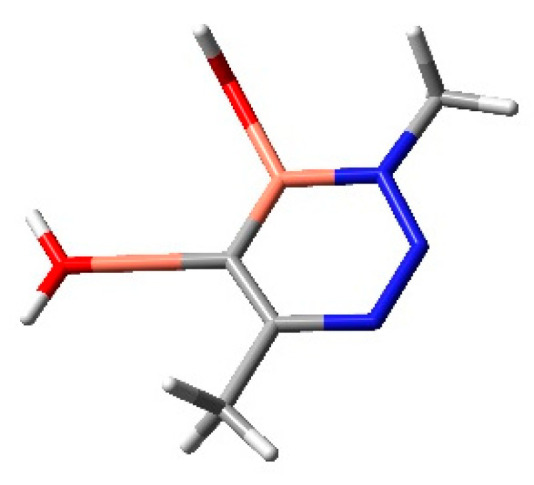
Six-membered cupracycle, intermediate of the reaction.

Instead, in the case of the catalyzed reaction, the metal is described through the LANL2DZ basis set. Firstly, the terminal alkyne binds to copper(I) as a π-ligand with a Cu6–N1 distance of 2.10 Å and an increase in acidity of its terminal alkyne proton. The second step consists of the binding of the distal nitrogen of the azide to the C-2 carbon of the acetylide with a barrier in water of 8.99 kcal/mol. In the corresponding TS, the C4-N3 bond length is 1.90 Å, while the Cu6–N1 becomes 2.02 Å. This is the rate-limiting step of the reaction for the Cu(I)-catalyzed process. From this passage, a six-membered Cupracycle is obtained (Figure 2). It is quite stable because of the absence of ring strain determined by the presence of two copper atoms and an sp^2^ hybridized carbon atom. Finally, the ring-closure process occurs with a barrier of 16.12 kcal/mol and a length of the C5–N1 forming a bond of 2.22 Å. As clearly evidenced, the coordination of the copper to alkyne changes the mechanism from a non-polar one-step to a polar stepwise with the preferred obtainment of a 1,4-cycloadduct.

In the same field, Hamlin et al. [20] investigated the cycloadditions of methyl azide with 2-butyne in comparison with cycloalkynes (cycloheptyne, cyclooctyne, cyclononyne). Calculations were performed at the M06-2X/6-311++G(d)//M06-2X/6-31+G(d) level. The ΔG^≠^ values are 16.5–22.0 kcal/mol lower in energy for cyclic adducts with respect to 2-butyne, which gives a less exergonic reaction. 

This study demonstrated that the higher reactivity of cyclic alkynes, in comparison with acyclic ones, can be attributed to three different factors. Surely, the first one is a reduction in strain or distortion energy, which is historically used to explain the acceleration of cycloaddition rate. However, the authors highlighted that it is accompanied by a smaller gap between HOMO and LUMO and a major orbital overlap, which is able to help the stabilization of orbital interactions. The smaller HOMO–LUMO gap is attributable to a stabilization the cycloalkyne π-LUMO, while the higher orbital overlap is probably due to a polarization of both the π-HOMO and π-LUMO lobes on the external π-face, pointing to the azide frontier molecular orbitals. 

A series of Cu complexes with N-heterocyclic carbene were prepared by Lin et al. [21] and used for the copper-catalyzed azide–alkyne cycloaddition reaction. The activities of the Cu complexes that resulted were related to the nature of the counterion X^−^ (I > Br > Cl∼BF_4_ > PF_6_) and to the rank of the trans effect. Experimental evidence allow for hypothesizing that firstly, the NHC ligand dissociated from the Cu and deprotonated phenylacetylene, giving (NHC-H)^+^ and phenylacetylide. Then, phenylacetylide and Cu bonded, forming (NHC-H)^+^[(PhC≡C^−^) CuX]^−^.

DFT calculations, performed at the M06/Def2-SVP level and using the C-PCM model for a solvent, suggest a mechanism characterized by the obtainment of acetylide copper halide as a consequence of NHC ligand dissociation and phenylacetylene deprotonation. After cycloadditions of methyl azide with mononuclear copper acetylide give a six-membered metallacycle, the reaction with the second copper catalyst occurs, forming an intermediate. This step is followed by ring contraction, leading to a dinuclear intermediate with higher stability.

In a second step, the six-membered metallacycle, achieved by the cycloadditions of methyl azide with mononuclear copper acetylide, reacted with the second copper catalyst, (NHC)CuX, to form the dinuclear intermediate, which, undergoing a quick ring contraction, form another more stable dinuclear intermediate. Finally, triazole is acquired after the NHC−H^+^ protonation. Moreover, calculations confirm that the NHC dissociation should occur in the first phase and not in the X^−^ dissociation.

**Scheme 1:**
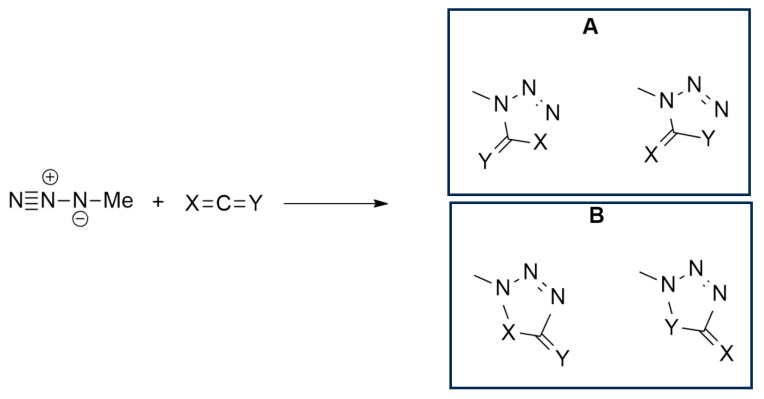
Possible products ((**A**) = 1,5-adducts; (**B**) = 1,4 adducts) of the 1,3-dipolar cycloaddition of methylazide with linear allenes (X, Y = CH_2_, NH, O).

Yu et al. [22] studied cycloadditions involving methyl azide and various allenes (propadiene, ketenimine, ketene, carbodiimide, isocyanic acid, and carbon dioxide), investigating reactivity, site- and regioselectivity through DFT calculations using a BP86 functional and TZ2P basis set. The formation of two possible regiospecific cycloadducts, the 1,5- or the 1,4-adduct, was taken into consideration and, in all cases, the formation of 1,5-adducts (Scheme 1A) was kinetically and thermodynamically favored over that of 1,4-adducts (Scheme 1B).

In the case of asymmetric heteroallenes, methyl azide prefers to attack the most electropositive terminal atom. Only in the case of ketene is the barrier for the attack to CO a little bit lower than that for the attack to CC (19.2 vs. 20.0 kcal/mol). With the increasing of the heteroatoms number, the process is less reactive (passing from CCC to CCN or CCO, from CCO to NCO or OCO, and from CCN to NCN). Cyclic allenes were also considered as cycloadducts. In these cases, the higher predistortion of cyclic allenes determines higher reactivity, connected with lower activation energies for the cycloaddition, because of a smaller HOMO–LUMO gap and thus the presence of more stabilizing orbital interactions.

**Scheme 2:**
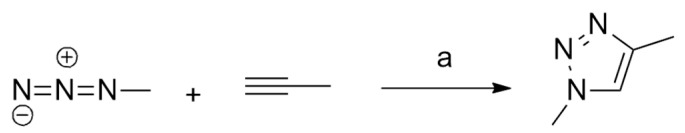
Reaction conditions: (a) Ag(I), AgCl/H_2_O.

Ben El Ayouchia et al. [23] investigated, through a combination of experimental and molecular electron density theory (MEDT) studies, the [3 + 2] cycloaddition reaction between methyl azide and propyne. Experimentally, the reaction, catalyzed by Ag(I), is fast at room temperature and the final product, 1,4-dimethyl-1,2,3-triazole, is obtained in good yields and easily isolated without any purification (Scheme 2).

Calculations were performed at both the B3LYP/6-31G(d,p) (LANL2DZ for Ag) and the ωB97XD/6-311G(d,p) (LANL2DZ for Ag) levels, choosing the water and chloride anion as ligands.

In the case of the uncatalyzed reaction, the cycloaddition occurs with high barrier and poor regioselectivity, giving both 1,4- and 1,5-dimethyl-1,2,3-triazoles. Instead, considering the Ag-catalyzed reaction, the 1,4-adduct is estimated to be regioselectively obtained, as experimentally verified.

**Scheme 3:**
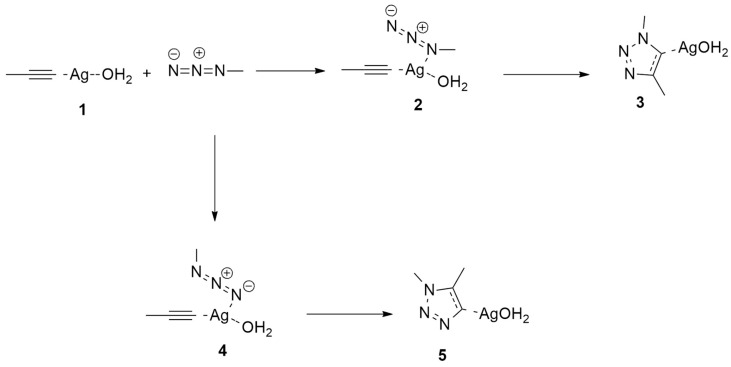
Ag(I)-catalyzed azide-alkyne cycloaddition involving catalytic species **1**.

**Scheme 4:**
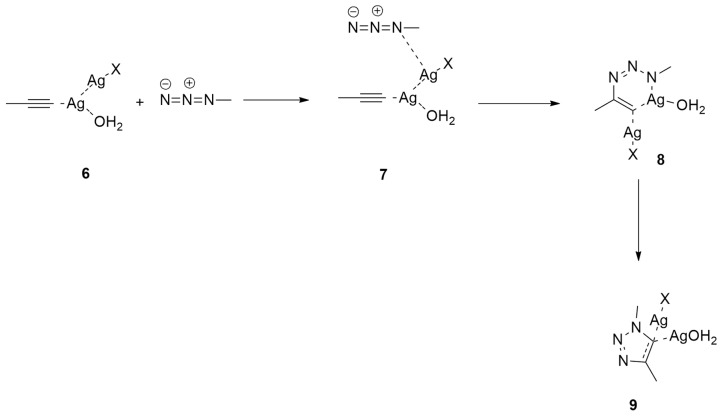
Ag(I)-catalyzed azide–alkyne cycloaddition involving catalytic species **6** (X = Cl, OH_2_).

Three different possible catalytic species are supposed (Scheme 3 and Scheme 4).

The reaction involving intermediate **1** is characterized by a single step, as shown in Scheme 3. The other two species, **6** (X = Cl, H_2_O), follow a two-step mechanism that has no experimental impact because of the endothermic nature of the corresponding intermediate complexes **8** (Scheme 4). 

In both cases, the rate-determining step is the nucleophilic attack of the N1 nitrogen atom of azide to the substituted C atom of propyne without energetic differences between chloride or water ligands.

This dinuclear route presents lower barrier values (about 4 kcal/mol) with respect to the mononuclear pathway. It is noteworthy that a similar stepwise mechanism was found using the two different functionals (B3LYP and ωB97XD), confirming the accuracy of B3LYP for the study of these cycloadditions.

**Scheme 5:**
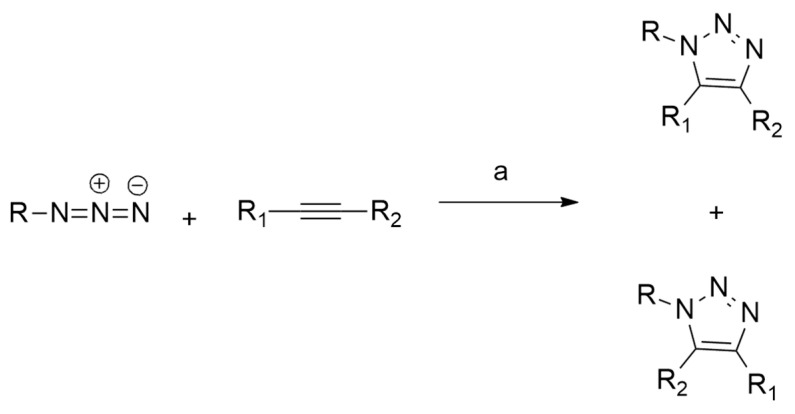
Reaction conditions: (a) Cp_2_Ni (10 mol %), Xantphos (10 mol %), Cs_2_CO_3_ (1 eqv), DCM, r.t., 24 h.

Kim et al. [24] performed a [3 + 2] cycloaddition of alkynes, cyanoalkynes, thioalkynes, and ynamides with differently substituted azides in the presence of nickel as a catalyst (Scheme 5). The products were obtained with significant regio- and chemoselectivity. In the case of cyanoalkynes, the 1,4,5-trisubstituted triazoles with the cyano group at position 4 of the ring are preferred. Instead, the thioalkynes and the ynamides showed an inverse regioselectivity leading to 1,4,5-trisubstituted triazoles with thiol and amide groups at position 5, respectively.

In order to clarify the reaction mechanism, a computational study was performed at the M06/6-31G**/cc-pVTZ(-f) level.

**Scheme 6:**
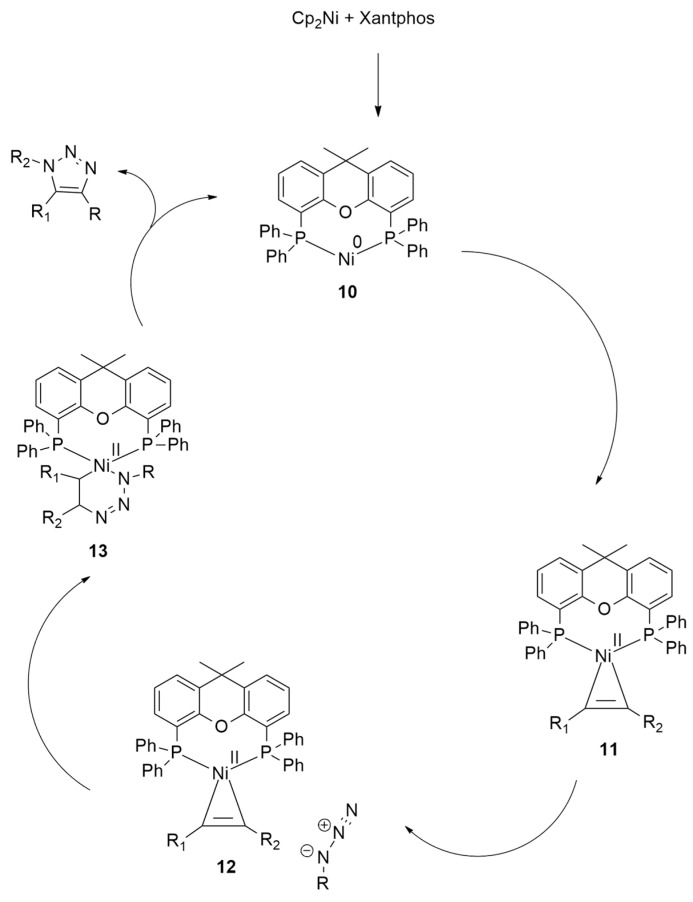
Computed reaction mechanism of nickel-catalyzed cycloaddition.

The computed mechanism, reported in Scheme 6, allowed for hypothesizing the formation of a nickelcyclopropene intermediate obtained by the oxidative addition of the alkyne substrate to the Ni(0)–Xantphos catalyst. Then, this intermediate gave the coupling with azide determining the regio- and chemoselectivity.

In detail, a Xantphos ligand forms a 14-electron Ni(0)–d10 complex **10** that, in turn, gives an exergonic oxidative addition of the alkyne. In this way, Ni(II)-cyclopropene intermediate **11** is afforded. Then, the interaction with azide allows the obtainment of **12**. Finally, the cycloaddition occurs, giving **13**, overcoming a barrier of 24.8 kcal/mol. 

This cyclization step is the rate-determining one of this nickel-catalyzed reaction on which regioselectivity depends. 

**Scheme 7:**
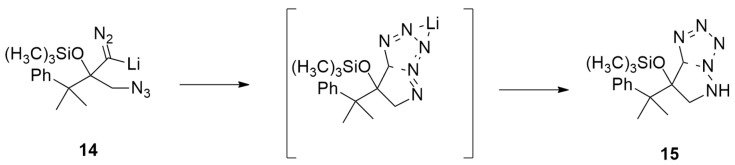
Dipolar cycloaddition between lithiated trimethylsilyldiazomethane and α-azido ketones.

Chen et al. [25] obtained bicyclic tetrazoles from the anionic [3 + 2] dipolar cycloaddition between lithiated trimethylsilyldiazomethane and α-azido ketones. DFT calculations were performed at the M06/6-31+G level, highlighting that the first step of the reaction is the chelation of the nitrogen-based dipole and dipolarophile by a lithium ion (Scheme 7).

The steric hindrance of groups bearing by the α-azido ketones affect the selectivity and efficiency of the cycloadditions in competition with the formation of triazines due to the C−H insertion of alkylidene carbene.

**Figure 3:**
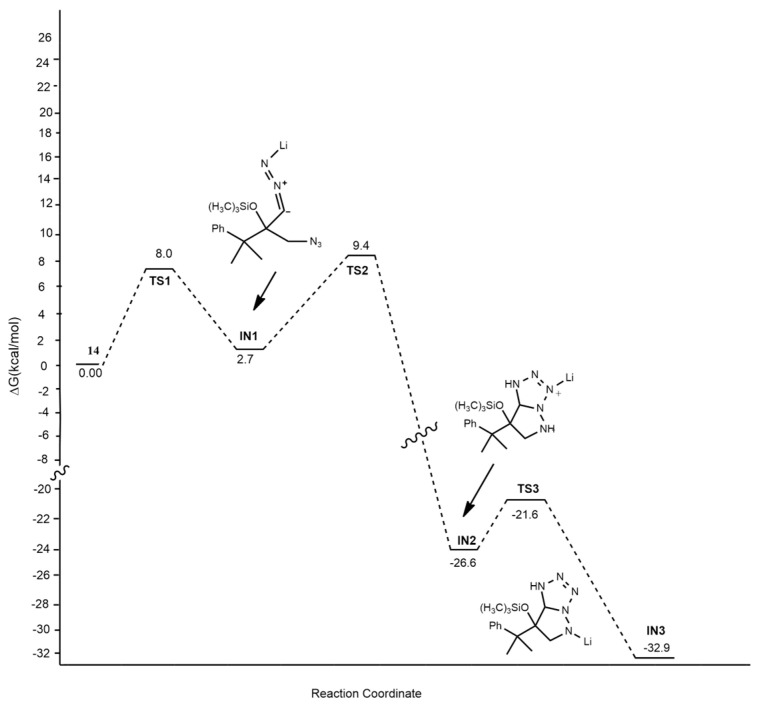
Energy profile of reaction leading to **15**.

The mechanism (Figure 3) starts from **14**, which is derived from lithiated trimethylsilyldiazomethane and α-azido ketones. It rearranges through a barrier of 8.0 kcal/mol to **IN1**. This intermediate undergoes the anionic [3 + 2] dipolar cycloaddition, passing **TS2** (9.4 kcal/mol) and leading to cycloadduct **IN2**. Then, **IN2** rearranges to the most stable aromatic intermediate **IN3** that is protonated, furnishing tetrazoles **15**. 

**Scheme 8:**
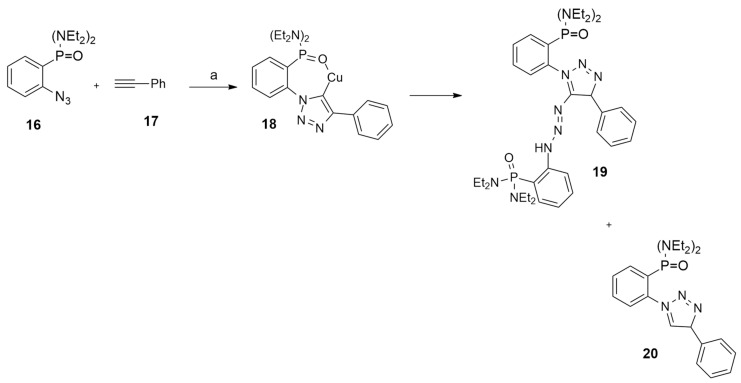
Reaction conditions: (a) CuSO_4_·5H_2_O/5 Na^+^ L-Asc. 1 eq, DMF (1 mL), t = 42 min, 25 °C, air.

Navarro et al. [26] synthesized 1,4-(disubstituted)-5-triazenyl-1,2,3-triazoles using a ligand-free domino copper(I)-catalyzed azide−alkyne−azide process, involving chelating aryl azides with polar groups in ortho to the azide moiety (i.e., P(O)(NEt_2_)_2_) and terminal alkynes (i.e., phenylacetylene **17**) (Scheme 8).

From this model reaction, fully substituted 1,2,3-triazole **19**, chemo- and regioselectively containing a triazenyl moiety, is obtained. Experimentally, a competition with a reaction route that generates the conventional click 4-(disubstituted)-triazole **20** was revealed. In order to clarify the process, DFT calculations were performed at the M06/6-311+G(d,p)-SDD/SMD(DMF)//M06/6-31+G(d)-SDD/SMD (DMF) level. Modeling data showed that chelation of the copper cation of the Cu-triazolide intermediate **18** by the ortho-substituted azide is crucial to avoid competition with the formation of conventional triazole **20**.

**Figure 4:**
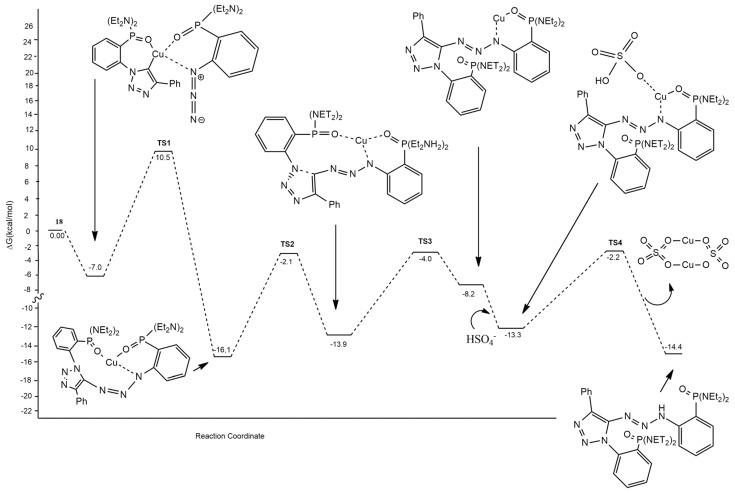
Computed pathway for the formation of **19** and **20**.

The mechanism involving the formation of **19** presents four steps and thus four different transition states. The first one (**TS1**) corresponds to the nucleophilic attack of Cu-triazolide **18** to the azide **16**. Then, the cis/trans isomerization of the triazene moiety (**TS2**), the Cu-oxygen decoordination of one P=O (**TS3**), and the protonation of the Cu-triazene (**TS4**) occurred (Figure 4). **TS1** is the rate-determining step of the process and is 0.5 kcal/mol lower in energy with respect to the TS that from **18** leads to **20**.

**Figure 5:**
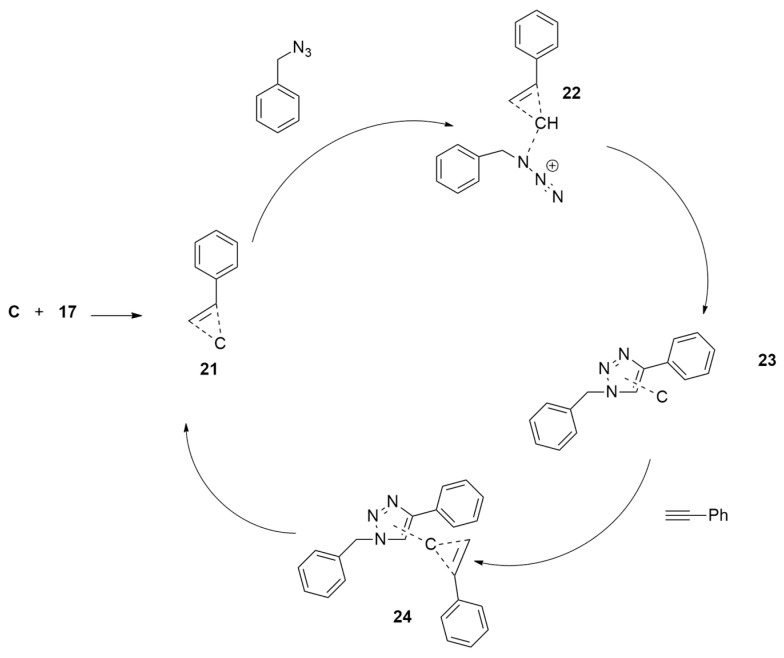
Catalytic cycle of CuAAC reaction of terminal alkynes.

Fang et al. [27] studied the copper(I)-catalyzed azide alkyne cycloaddition (CuAAC) using as the catalyst a supported carbon nanotube (CNT) Au_4_Cu_4_ cluster. The Au_4_Cu_4_/CNT system heterogeneously and efficiently catalyzes the CuAAC reaction of terminal alkynes without alkyne deprotonation to a σ,π-alkynyl intermediate, following the cycle reported in Figure 5.

DFT results showed that HC≡CPh was activated by π-complexation with Au_4_Cu_4_ forming the bimetallic σ, π-alkynyl intermediate that is the real catalyst of the reaction. The terminal H atom of the alkyne is not stripped during catalysis. This type of catalyst allows also the reaction involving internal alkynes. On the contrary, the Au_11_/CNT and Cu_11_/CNT nanocatalysts are not active for the last type of alkynes, highlighting the synergistic effects of Au and Cu in Au_4_Cu_4_.

Zu and Kinjo [28] investigated the regioselective cycloaddition involving 1,2-diboraallene **25**, showing cumulated C=B and B=B double bonds, and azide, without catalysis and using mild conditions, leading to diboratriazole **26**. 

**Scheme 9:**
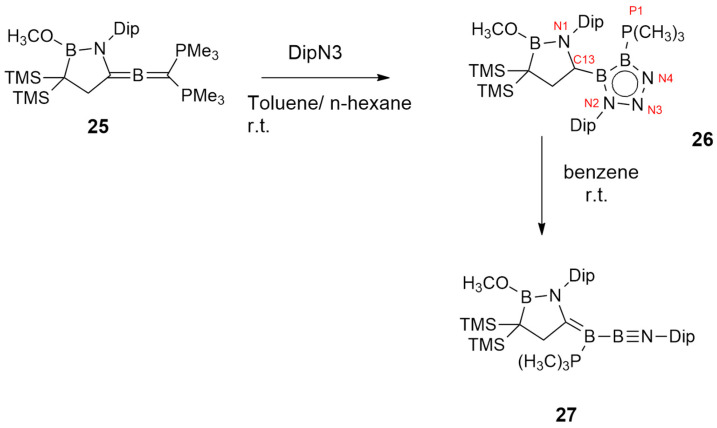
Synthesis of compounds **26** and **27**.

Compound **25** reacted with 2,6-diisopropylphenyl azide, giving cycloadduct **26** (Scheme 9). The same reaction, conducted with other azides (i.e., trimethylsilyl azide, 1-adamantyl azide, and phenyl azide), does not provide the same product, highlighting the importance of the azide electronic and steric factors for the reaction trend. 

Cycloadduct **26** presents low solubility and so it was successfully isolated. Nevertheless, it spontaneously releases N_2_, giving **27**. 

The electronic structure of **26** was elucidated calculating frontier orbitals through DFT methods at the B3LYP/6-311G(d,p) level. The HOMO orbital was dominated by the π-bonding orbital over the C13–B2–B3 moiety (see numbering in Scheme 9), while HOMO-1 included the *σ*-bonding of the B2N3 ring. The HOMO-2 orbital coincided with π-bonding orbitals B3–N4 and B2–N2, which were polarized toward the nitrogen. The LUMO orbital corresponds to the p orbitals on C13 and B3 and also includes the π-type orbital over the B-N moiety in cyclic (alkyl)(amino)carbene. 

**Figure 6:**
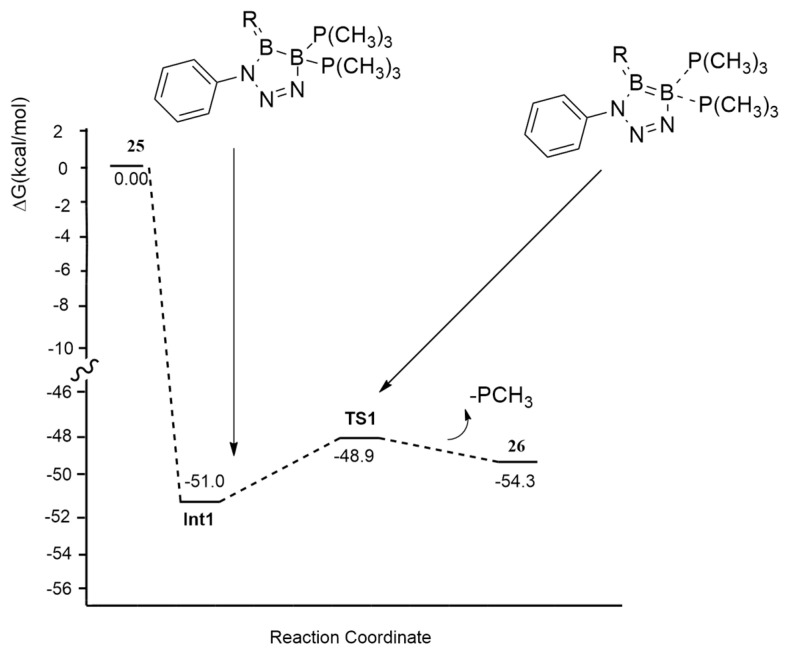
Reaction mechanism leading to **26**.

Moreover, the authors investigated the reaction mechanism leading to **26** (Figure 6). 

The phenyl azide attacks 1,2-diboraallene from the CH_2_ side. The initial coordination of two nitrogen atoms of azide with the two B atoms affords the five-membered ring **Int1** without overcoming any barrier. Passing through the low barrier **TS1**, of only 2.1 kcal/mol, the elimination of one PCH_3_ group occurs, giving **2**.

**Scheme 10:**
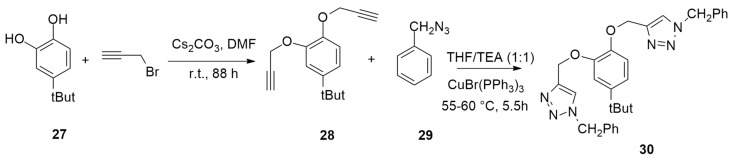
Synthesis of 1,2,3-triazoles through CuAAC.

Singh et al. reported [29] 1,2,3-triazole scaffolds, obtained through Copper(I)-catalyzed alkyne−azide cycloaddition (CuAAC) as a chemosensor probe (Scheme 10), which is able to recognize heavy metal ions, such as Pb (II) and Hg (II).

The structures of the ligand **30** and the complexes with Pb(II) and Hg(II) were optimized through DFT calculations at the B3LYP/6-311++G(d,p) level and B3LYP/LANL2DZ for metals. 

In the complex, the metal ion is coordinated to one of the N atoms of the 1,2,3-triazole ring. The FMO analysis showed that the HOMO is mostly delocalized over the 4-tertbutylcatechol moiety, while the LUMO orbital is delocalized over one of the arms bonded to the 1,2,3-triazole. The energy difference between HOMO and LUMO is lower for the complex (1.11 eV) with respect to the free ligand (5.203 eV). These 1,2,3-triazoles, synthesized via the CuAAC methodology, can be efficiently used for their ion-sensing properties.

In 2022, Chiavegatti Neto et al. investigated the different steps of metal-free [3 + 2] cycloadditions with enolates/enols and azides using DFT calculations [30].

**Scheme 11:**
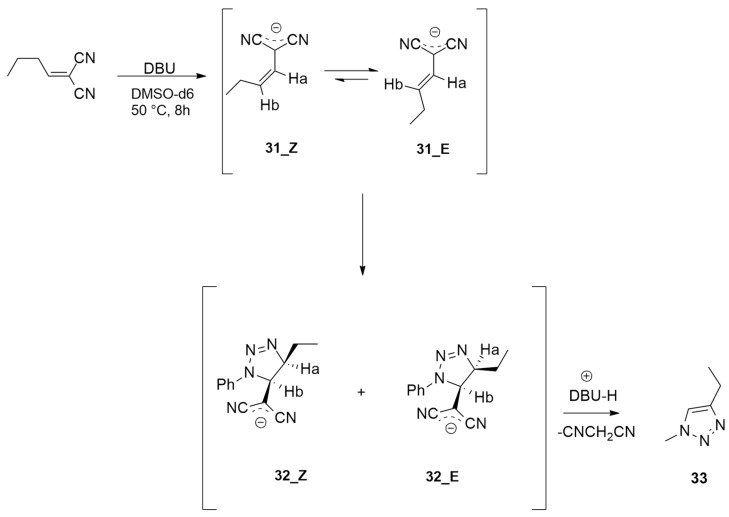
Synthesis of compound **33**.

Experimentally, the reaction was conducted starting from 2-butylidenemalononitrile DBU and PhN_3_ in DMSO-d6 at 50 °C (Scheme 11). After the obtainment of both Z and E isomers of **31**, PhN3 was added, leading to **Z-32** and **E-32.** Calculations were performed at the M06-2X/6-31+G(d,p) level starting from 2-propylidenemalononitrile, which was achieved from the Knoevenagel condensation between propanal and malononitrile. The following deprotonation gives intermediates **Z-31** and **E-31** that react with phenyl azide, passing the two barriers **E-TS1** and **Z-TS1**, the first one being slightly lower. Experimental data show that the trans isomers prevail. The irreversible cycloaddition leading to **E-32** and **Z-32** ends the final elimination, furnishing compound **33**. The reaction energy profile suggests that the elimination is the rate-determining step of the process with a higher energy with respect to the cycloaddition.

**Scheme 12:**
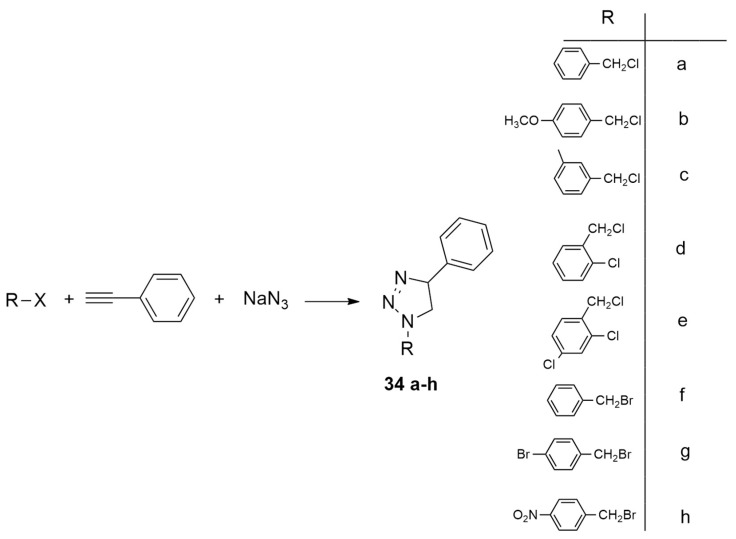
Cu(I)-catalyzed AAC reactions, leading to **34 a**–**h**.

On the basis of the growing interest toward economic viability and sustainability, attention was focused on the use of copper catalysts for coupling reactions. Gholivand et al. [31] reported the cinnamaldehyde-derived Schiff base ligand N,N′-bis (trans-cinnamaldehyde) ethylenediimine (C_20_H_20_N_2_, **L**) used to obtain Cu (I) complexes (i.e., [CuC_20_H_20_N_2_)PPh_3_Cl] (**C1**) and [Cu(C_20_H_20_N_2_)PPh_3_Br] (**C2**) and applied to the azide–alkyne click reaction (AAC) as catalysts (Scheme 12).

Experimentally, the best reaction conditions were shown to be 15 mol % catalyst, 45 °C, 30 min and water as the solvent. The structure and the reactivity of **C1** and **C2** were studied through quantum calculations using moduleDMol3 and the LDA/PWC level. The minimum energy conformations were located, and the FMO analysis was performed. Complex **C2** is more reactive than **C1**, and adducts are obtained faster using **C1** rather than **C2** as the catalyst, except in case a and b (Scheme 12).

**Scheme 13:**
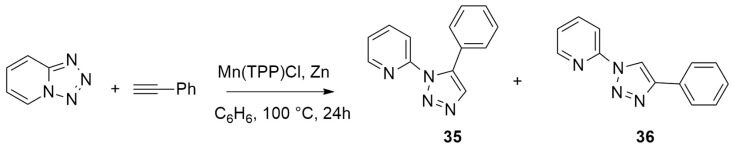
Synthesis of 1,5-disubstituted 1,2,3-triazole **35**.

Song et al. [32] used DFT calculations, starting from 1,2,3,4-tetrazole a Mn-catalyzed click reaction using phenylacetylene and obtaining 1,5-disubstituted 1,2,3-triazole **35** with complete control of the regioselectivity (Scheme 13). The authors proposed, on the basis of their results, a very interesting manganese-copper/zinc cooperative mechanism.

Optimizations were performed using the M06/6-311++G** basis set for all other atoms and the LANL2DZ basis set for metals.

The mechanistic analysis highlighted three different steps for the process: (1) the opening of the tetrazole; (2) a variation of the Mn coordination site; and (3) the 1,3-cycloaddition assisted by ZnCl_2_. This last step is the regioselectivity-determining one of the reactions.

**Figure 7:**
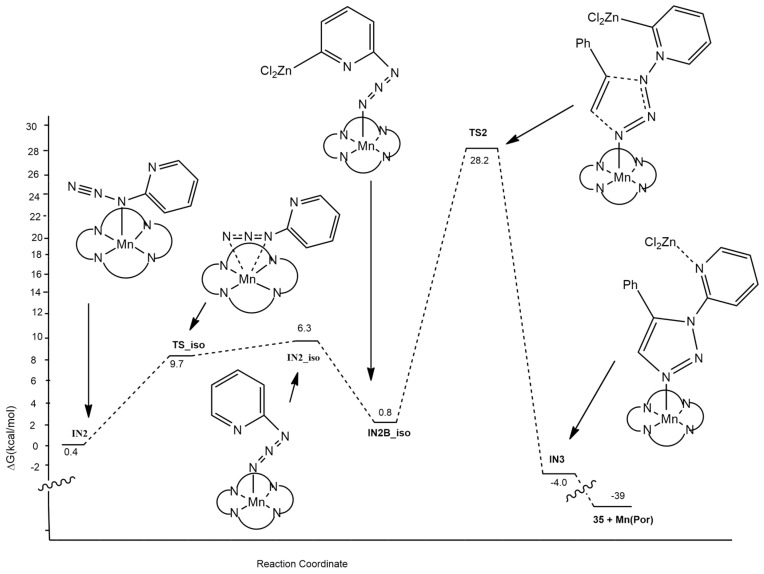
Calculated free energy profiles for the click reaction.

The reaction (Figure 7) starts with tetrazole ring opening giving complex **IN2**. Cycloaddition is not possible at this level because of the steric hindrance caused by the presence of pyridine on the N1 atom. The isomerization of **IN2** to **IN2_iso** occurs. At this point, ZnCl_2_, obtained in conjunction with the catalyst, coordinates to the N4 atom of **IN2-iso**, giving **IN2B-iso**. Then, the 1,3-cycloaddition leading to the formation of C1–N1 and C2–N3 bonds in the presence of ZnCl_2_ occurs, passing a barrier of 27.4 kcal/mol (**TS2**). Finally, the reaction furnishes the 1,5-disubstituted 1,2,3-triazole **35** and Mn(Por) catalyst regenerated. The authors considered also the possibility of a pathway without ZnCl_2_ participation; however, it was shown to be kinetically disfavored.

Fukuura and Yumara [33] investigated, through DFT calculations, the 1,3-dipolar cycloaddition reaction involving phenylacetylene and phenyl azide on the surface of carbon nanotubes, showing diameters in the range 10–14 Å. The authors used QM/MM ONIOM calculations, treating guest molecules with the B97D functional and host nanotubes with UFFs (universal force fields). The regioselectivity of the reaction was studied, paying attention to the formation of the 1,4- and 1,5-triazoles.

The nanotube support favors, both from the kinetic and thermodynamic point of view, the formation of the 1,4-triazoles as cycloadducts. Considering the 1,4-route, reactants are planar, because they are small with respect to the cavity of tubes. In this case, the nanotubes allow lower ΔE^≠^ without any dependence on their diameter values.

Conversely, in the 1,5-approach, the phenyls of cycloadducts overlap each other, giving a stacking interaction. This structure in thin tubes determines repulsive orbital interactions between the two rings, causing an increase in the activation energies with a decrease in the tube diameter.

#### 2.1.2. Nitrile Oxides

The interest in the chemistry of nitrile oxides is primarily due, when they react with alkenes as dipolarophiles, to the synthesis of isoxazoline derivatives, which are regarded as masked b-hydroxycarbonyl compounds that can be further converted into synthetically useful building blocks [34].

**Scheme 14:**
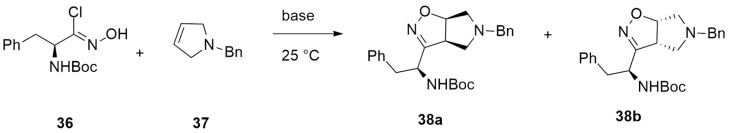
Nitrile oxide [1,3]-dipolar cycloaddition leading to **38a**,**b**.

Bucci et al. [35] reported the synthesis of tert-Butyl 1-(5-Benzyl-3a,5,6,6a-tetrahydro-4H-pyrrolo[3,4-d]-isoxazol-3-yl)-2S-phenylethyl)carbamate **38a**,**b** through a nitrile oxide [1,3]-dipolar cycloaddition between N-tert-Butoxycarbonyl [1-Chloro-1-(hydroxyimino)-3-phenylpro-pan-2-yl)]carbamate **36** and N-benzyl-3-pyrroline **37** and base (Scheme 14). 

From this reaction, two diastereoisomers can be formed, such as the Δ2-isoxazoline derivatives **38a** and **38b**.

In order to optimize the yield and control diastereoselectivity, different reaction conditions were tested. The use of more polar solvents allows for better yields when a weak organic base, with a pKb value in the range 5–3, is used. Also, inorganic bases and MeCN as the solvent give interesting results. 

By-products can be formed consequently to the rate of formation of nitrile oxide.

The diastereoisomeric ratio is influenced by the choice of the organic or inorganic base, moving from a 1:3 ratio in favor of adduct **38b** to a 1:1 ratio, respectively.

A complete conformational search of **38a** and **38b** was performed through MM calculations. The most stable geometries were optimized using DFT methods at the CPCMHCTC/6-11+G(d,p)//HCTH/6-31+G(d) level and MeCN as the solvent and C-PCM method. From the two conformations, the transition states leading to 3a and 3b were located. The reaction was shown to be under kinetic control, and the predicted ratio for **38a** and **38b** is 28:72, being a value very similar to that that experimentally determined.

The transition state **TS-3b** is stabilized by a T-shaped CH/π interaction involving the two aromatic rings. Conversely, in **TS-3a**, an interaction between the two phenyls is substituted by a CH/π interaction between the Boc group and the pyrroline benzyl with a slightly lower stabilization.

The authors conclude that the use of an organic base avoided π interactions between the aryls of pyrroline and nitrile oxide, influencing the diastereoselection of the cycloaddition.

**Scheme 15:**
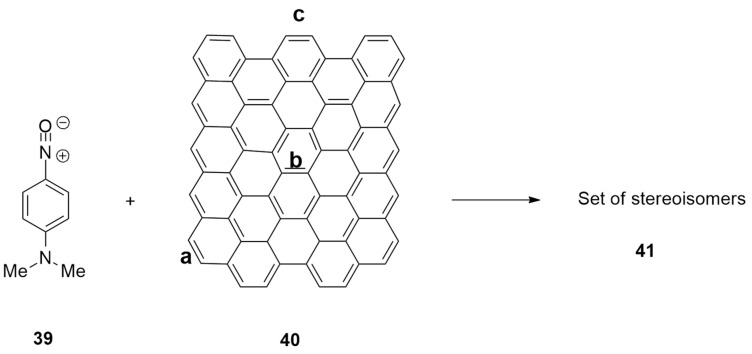
Cycloaddition of graphene **40** and nitrile oxide **39**.

The first cycloaddition of nitrile oxide **39** to the graphene sheets **40** was described both theoretically and experimentally by Uceta et al. [36]. The authors performed DFT studies of the interaction between graphene **40** and nitrile oxide **39**, highlighting the practicability of 1,3-dipolar cycloadditions leading to the isoxazoline ring (Scheme 15). Calculations were performed at the (U)M06-2X level and using the 6-31+G(d,p) basis set.

Modeling data showed that the DE between HOMO and LUMO is not high, and the reaction can occur. The reaction of graphene with **39** is a normal electron demand cycloaddition, involving the HOMO of nitrile oxide and the LUMO of graphene.

The aromatic ring of compound **39** can approach graphene in two ways, i.e., to the armchair side (r1) or at the zigzag side (r2). Moreover, the aromatic moiety can be placed ahead (c1), behind (c2), or above (c3) with respect the graphene sheet, giving six possible “approximations”.

The approach r2 is kinetically and thermodynamically preferred because of the CH–π stabilizing interaction between **39** and graphene **40**. The preferred approaches pass through the same TS that presents the aromatic group parallel to the graphene, minimizing π–π interactions and leading to three adducts. The favorite ones were those with the lower steric interactions with graphene (r2c1 and r2c3). Nevertheless, data highlight that nitrile oxides are less reactive than nitrile imines, whose cycloaddition to the graphene sheets was previously reported in the literature [37]. 

**Scheme 16:**
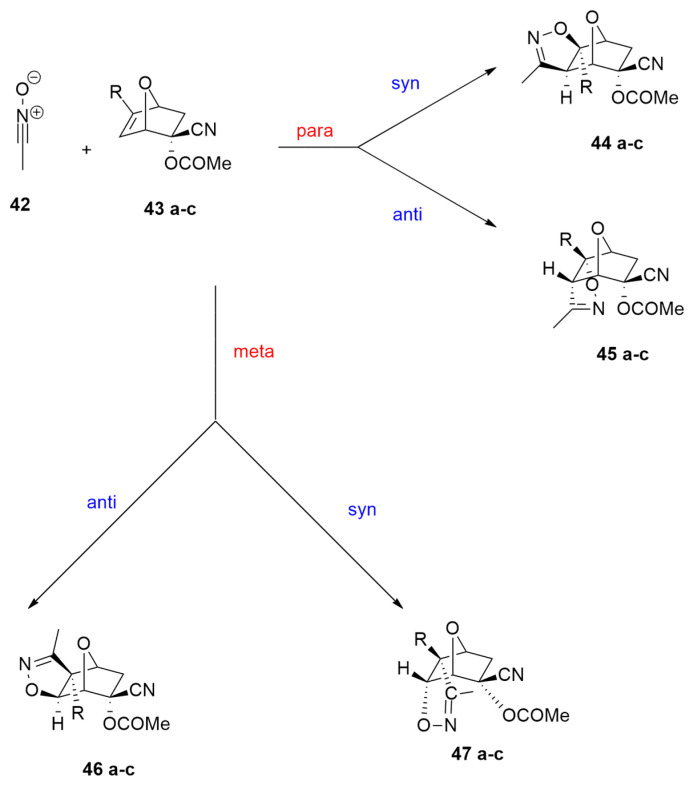
Different regioisomeric and diastereofacial routes of reaction between **42** and **43 a**–**c**.

The 1,3-dipolar rearrangement involving acetonitrile oxide **42** and (*1S*,*2R*,*4S*)-2-cyano-7-oxabicyclo[2.2.1]hept-5-en-2-yl acetate derivatives **43a**–**c** was studied, and a BET (bonding evolution theory) analysis was conducted [38]. Optimizations were performed at the ωB97X-D/6-311G(d) level of calculations. For this reaction, four different reaction channels can be investigated. In fact, nitrile oxide **42** reacts with the oxanorbornenic compounds **43a**–**c** through *syn* or *anti*-attacks and para and meta regioisomeric approaches of nitrile oxide on 7-oxanorbornenic derivatives (Scheme 16). 

The para routes are preferred with respect to the meta ones because of the most favorable electrophile–nucleophile interaction involving the O1 of 1 and the C5 of **43a**–**c**. This lowers the activation barriers along the para channels. Moreover, a syn attack presents TSs as more stable than those of the *anti* way. In fact, the *syn* route benefits from an interaction between the partial positive charge on the N atom of **42** and the partial negative charge on the bridge oxygen of oxanorbornenic derivative. In addition, a steric hindrance, due to the proximity of the methyl group of **42** and that of acetate, disfavors the *anti* attack. In conclusion, the *syn* isomers are preferred both from a kinetic and a thermodynamic point of view. 

The final BET analysis on the syn reaction channel showed that the formation of the C−C bond occurs before the formation of the O−C one through a usual sharing model. The topological changes along the reaction pathway take place in a highly synchronous way.

In order to control hyperglycemia that causes type 2 diabetes, glycogen phosphorylase (GP) inhibitors were prepared [39]. Spiro-oxathiazoles were demonstrated to be potent GP inhibitors and were obtained through a one-step 1,3-dipolar cycloaddition between an aryl nitrile oxide and a glucono-thionolactone.

**Scheme 17:**
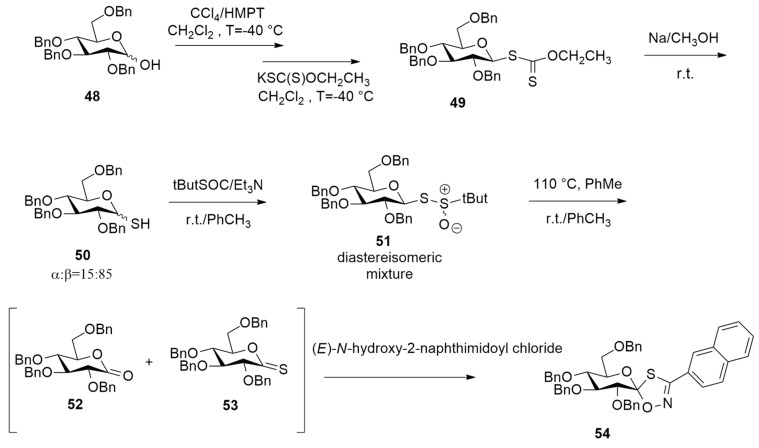
Synthesis of 1S-glucopyranosylidene-spiro-oxathiazole **54**.

As reported in Scheme 17, hemiacetal **48** was treated firstly with CCl_4_ and HMPT and then with potassium O-ethyldithiocarbonate at −40 °C, leading to dithiocarbonate **49**. The following methanolysis gives **50**, which in turn reacts with tert-butyl-sulfinyl chloride, affording β- thiosulfinate **51** as a diastereoisomeric mixture.

The subsequent thermal elimination conducted in toluene under reflux gives thionolactone **53** and D-gluconolactone **52** as a by-product. D-glucono-δ-thionolactone **53** was a dipolarophile of the 1,3-dipolar cycloaddition, synthesizing **54** with excellent regio- and stereoselectivity. The authors found that the rate-determining step of the reaction was the step from **51** to **53**. So, a DFT mechanistic study of the thermolysis was performed at the WB97XD2/6- 311G(d,p) level and considering a solvent (toluene) with the PCM method and SMD solvation model. Calculations evidenced that the thermal elimination step occurs, passing a five-membered transition state and involving the anomeric proton. This mechanism benefits from an anomeric effect and furnishes thionolactone **53** and tert-butyl sulfenic acid.

**Scheme 18:**
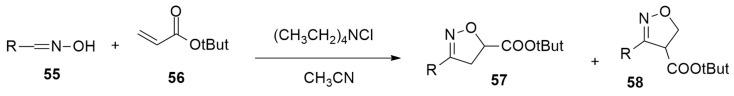
1,3-dipoalr cycloadditions leading to isoxazolines.

Holman et al. [40] obtained substituted isoxazoline exploiting an electrochemical method. This technique shows short reaction times and results to be green because they are characterized by minimal waste generation and the absence of toxic or expensive oxidizing reagents. The model reaction is reported in Scheme 18.

The chlorination of oxime **55** is electrochemically achieved by the oxidation of chloride anions to chlorinating electrophilic form. The in situ formation of nitrile oxide is followed by an electrochemically enabled regio- and diastereoselective reaction with electron-deficient alkenes, giving the desired products. For this reaction, both aromatic and alkyl aldoximes can be used.

**Scheme 19:**
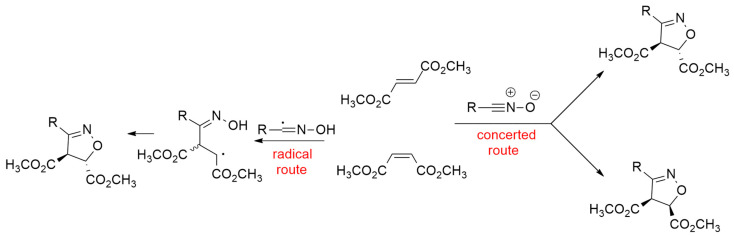
Different proposed mechanisms for the [3 + 2] cycloaddition between nitrile oxide and alkenes.

The mechanism of the reaction was studied through DFT calculations at the M06-2X/Def2TZVP/SMD(MeCN) level. Two different pathways, reported in Scheme 19, were investigated: (A) stepwise radical-mediated and (B) a concerted [3 + 2] cycloaddition.

**Figure 8:**
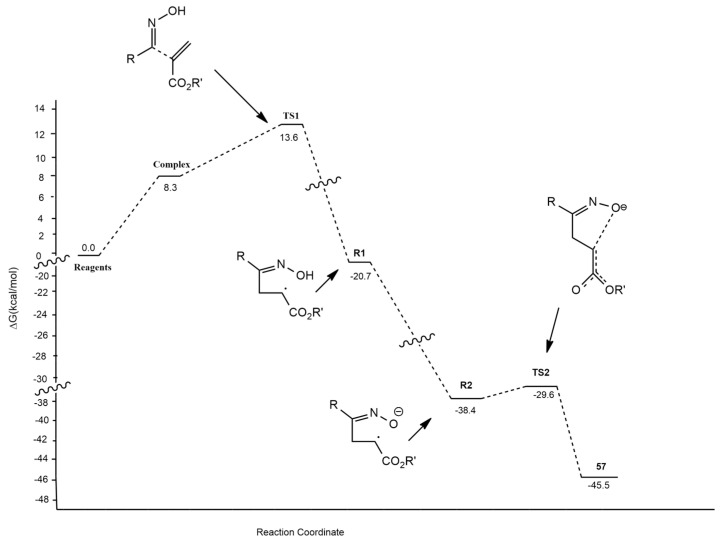
Free energy profile of the calculated open-shell radical mechanism leading to isoxazolines **57**.

All modeling data are consistent with the A pathway (radical mechanism), whose barriers were 2.5 times lower in energy than those of the B pathway. Also, the determined regioselectivity and the formation of the 3,5-isoxazoline confirms the radical mechanism reported in Figure 8.

**Scheme 20:**
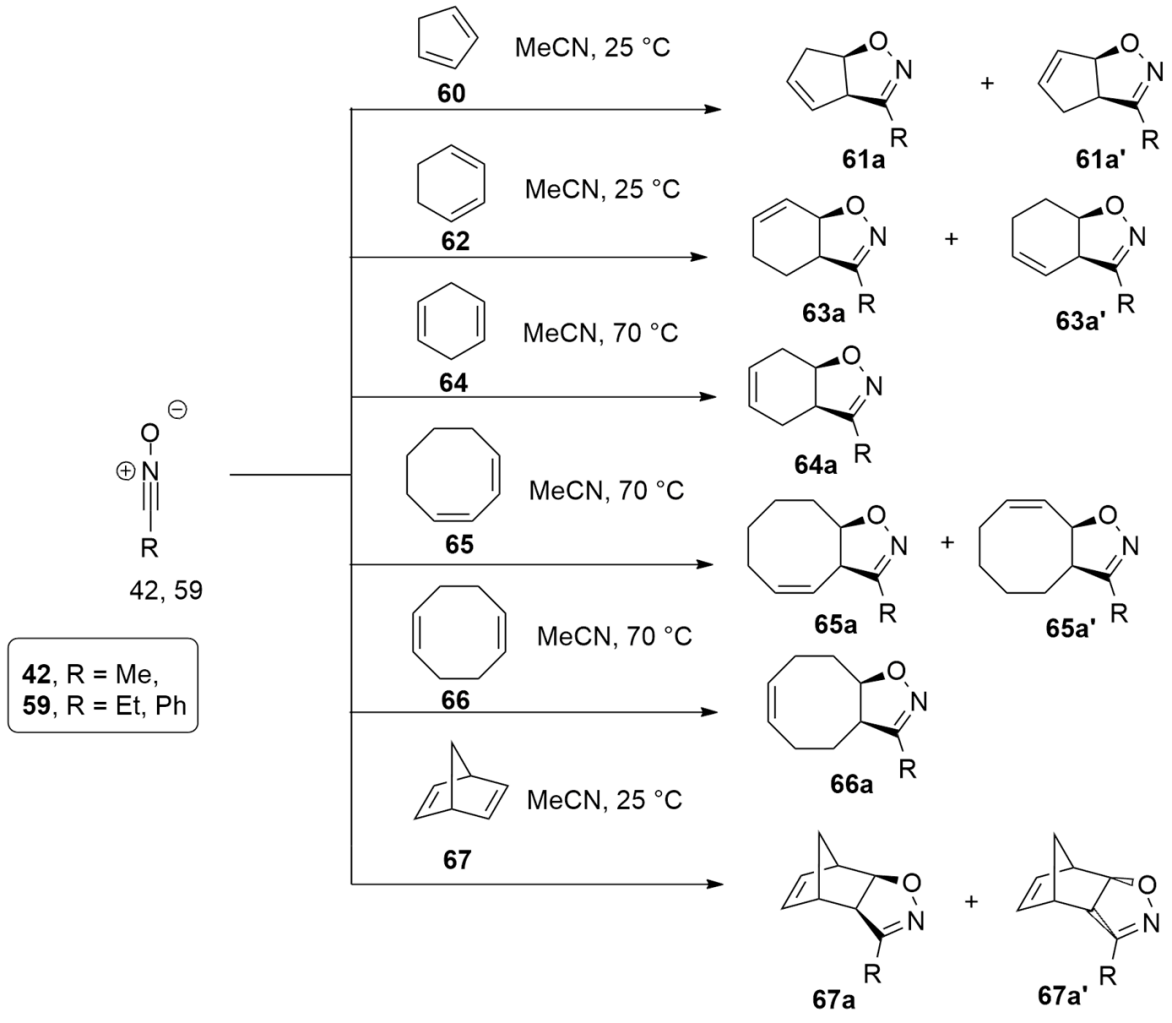
The 1,3-dipolar cycloadditions of **42**, **59** with cyclic dienes **60**, **62**, **64**, **65**–**67**.

The 1,3-dipolar cycloaddition of nitrile oxides with cyclodienes leading to cycloalkene-fused isoxazolines then converted into dialkenylated heterocycles through ring opening and cross-metathesis was studied at the M06-2X/6-311+G(d,p) level of theory (Scheme 20) [41].

Calculations allowed for locating the different TSs, giving the adduct with the O atom (named a) or the C-atom (named a′) of the isoxazoline closer to the C-sp^2^ of the cycloalkene ring. The determined relative activation free energies are in very good agreement with the product ratios experimentally obtained. In fact, in case of **60**, the TS of route a is more stable at about 2.7 kcal/mol, and the datum is confirmed by the experimentally found regioselectivity. The diene **62** showed lower barriers and consequently higher reactivity. Moreover, the product from via a is kinetically favored, while that from via a’ is thermodynamically preferred. Conversely, the reactions with **64**–**67** present very high barriers, and also experimentally, products are not yielded. Using an activation strain model, the authors reveal that the distortion energies of the nitrile oxide determine the regioselectivity. 

Table 1, reported below, summarizes the main findings (i.e., reagents, reaction conditions, catalysts, and level of calculations) of the papers focused on 1,3-DC involving 1,3-dipoles of propargyl-allenyl type.

### 2.2. 1,3-Dipoles of Allyl Type

Allyl-type 1,3-dipoles are bent and contain only one double bond in a canonical form. 

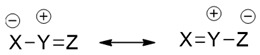



Some of these are here shown (V–VIII).

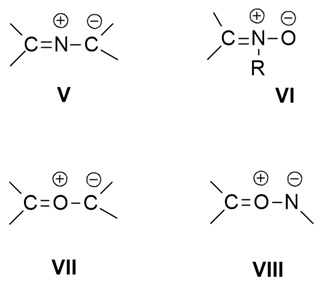



In the following sections, the cycloaddition reactions involving the azomethine ylides (V) and nitrones (VI) will be discussed.

#### 2.2.1. Azomethyne Ylides

Azomethine ylides have been employed in many elegant applications for the preparation of a pyrrolidine nucleus and for the productions of natural products, drugs, and agrochemical compounds [42].

**Scheme 21:**
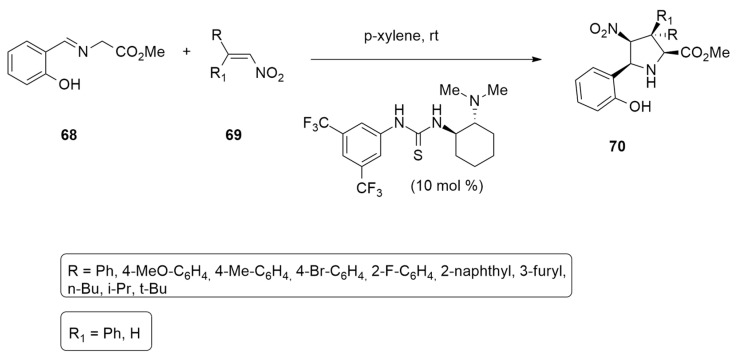
Organocatalytic synthesis of tetrasubstituted pyrrolidines.

The organocatalytic reaction of ortho hydroxy imine **68**, with nitro alkanes **69** in the presence of N,N′-bis[3,5-bis(trifluoromethyl)phenyl]-thiourea as catalyst, has been used to prepare a series of tetrasubstituted pyrrolidines **70** in high yields, high enantiomeric excess and excellent *exo/endo* selectivity (Scheme 21) [43]. The 1,3-dipolar cycloaddition process occurs because of the ortho hydroxy group present in phenyl ring of **68**, that, via an Intramolecular hydrogen bond, increases the acidity of the proton in the α-position of the imine group and makes the formation of the azomethine ylide possible even if the starting imine contains only one activating group. 

A mechanistic study of the model reaction between 1-phenyl-2-nitroethene **69** with imine **68** and in presence of the catalyst reported in Scheme 21 was performed using DFT methods at the M06-2X/6-31g(d,p) level. Calculations reveal the ability of the hydroxy group to activate the dipolar cycloaddition reaction. The formation of two different hydrogen bonds was shown to be essential for reactivity, i.e., there was an intramolecular hydrogen bond of the azomethine ylide in equilibrium with the starting imine and an intermolecular one with the catalyst.

**Figure 9:**
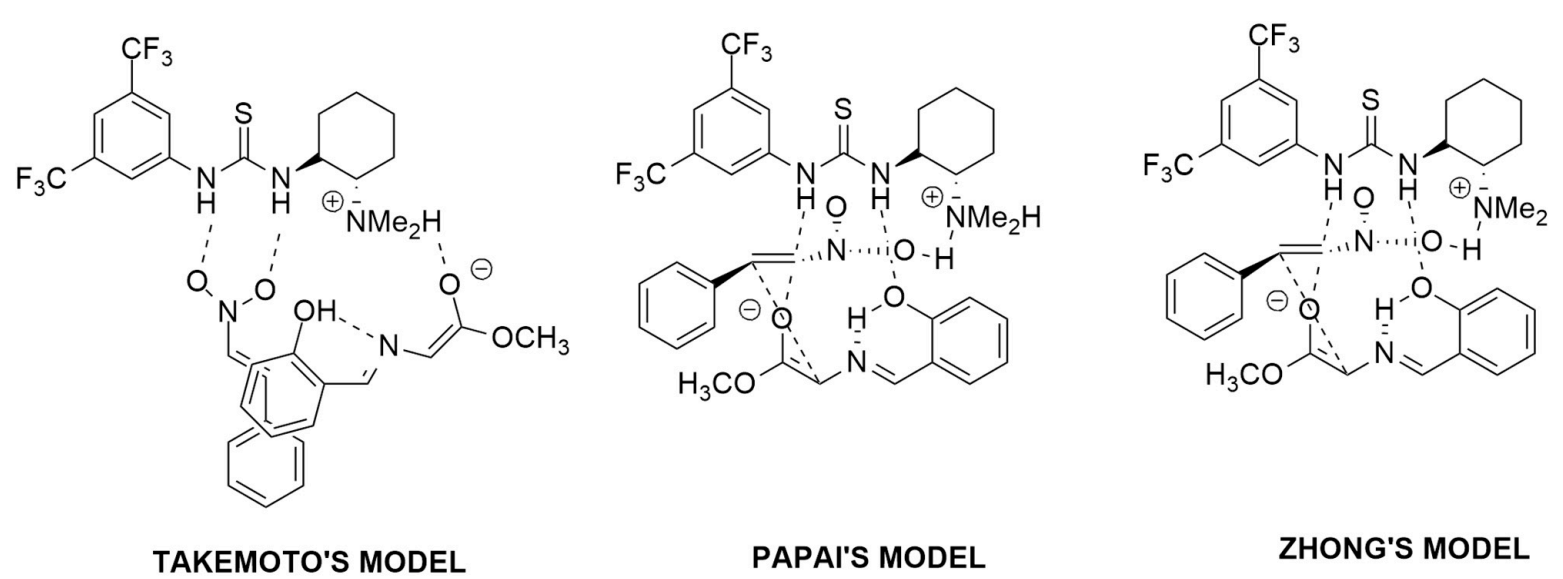
Pre-association complex intermediates for Takemoto, Papai and Zhong models.

In detail, during the study, the authors considered for the reactants three different coordination points with the catalyst taking into account both the *endo* and *exo* approaches and considering different models (Takemoto, Papai and Zhong). In all the cases, the pre-association complex intermediates and the corresponding TSs were located. In Takemoto’s model, the nitroalkene is directly coordinated to the catalyst thiourea, while in Papai’s one, the same moiety is connected to the azomethine ylide. Finally, in Zhong’s model, an additional hydrogen bond of the aryl group of the catalyst with the azomethine ylide is detected (Figure 9).

Zhong’s model was shown to be the favorite one with a barrier of only 14.48 kcal/mol to pass from the intermediate to the product.

**Scheme 22:**
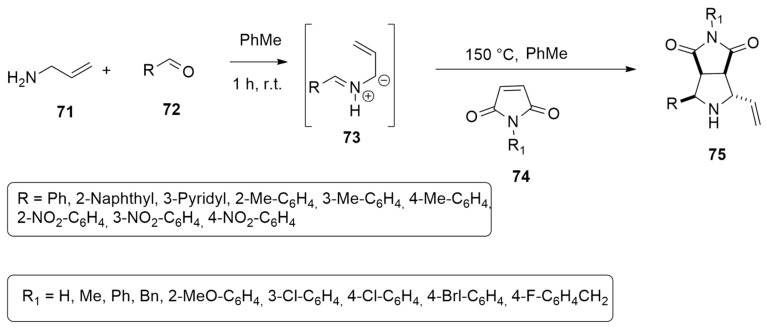
Synthesis of bicyclic pyrrolidines **75**.

Selva et al. [44] successfully developed a two-step procedure leading to bicyclic pyrrolidines **75** in moderate to good yields (31–70%). They described as key intermediates non-stabilized azomethine ylides **73**, obtained by the condensation of allyl ammine **71** with aldehydes **72** followed by the addition of maleimides **74**, in toluene at 150 °C (Scheme 22). 

**Scheme 23:**
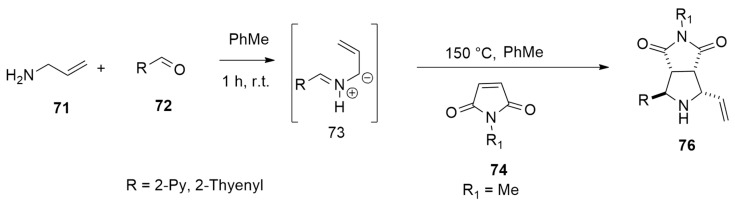
Synthesis of *exo*-2,5-trans adducts **76**.

The reaction occurs with high diasteroselection affording, as major adducts, the *endo*-2,5-trans derivatives **75** (Scheme 22). However, an inversion of diastereoselectivity was observed with 2-pyridinyl and 2-thienyl substituents, where the major products were the *exo*-2,5-trans adducts **76** (44–47% yield, respectively) (Scheme 23). 

**Scheme 24:**
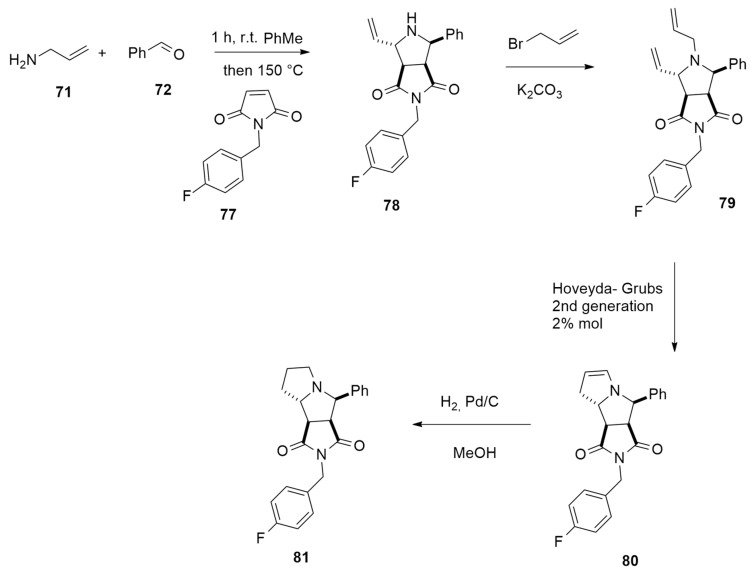
Synthesis of thrombin inhibitor **81**.

The above methodology was applied to the diastereoselective synthesis of tricyclic thrombin inhibitor **81** in 47% yield, starting from allyl amine **71**, benzaldehyde **72** and N-(4-fluorobenzyl)maleimide **77** according to Scheme 24. 

In order to rationalize the observed diastereoselectivity, a complete DFT study was performed through optimizations, employing the B3LYP functional in conjunction with Grimme’s dispersion correction and the def2SVP basis set. The authors considered the different possible conformers of the ylide able to stabilize the negative charge at either the allylic or the benzylic position. The formation of the ylide was shown to be the rate-limiting step of the process.

**Scheme 25:**
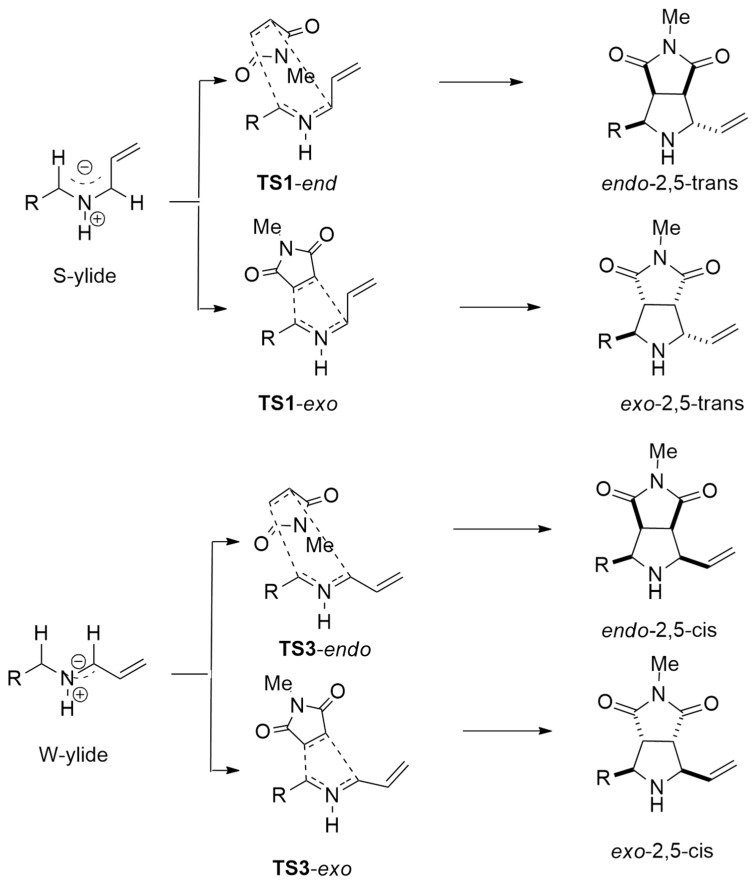
Located TSs for *endo* and *exo* routes, starting from S-ylide and W-ylide.

Studying the different *endo* and *exo* approaches, the TSs were located and the S-ylide formed the fastest with respect to the most stable W-ylide (Scheme 25). 

For the cycloaddition reaction, the *endo* route has lower barriers with respect to the *exo* one. Considering the benzaldehyde-derived ylides, a difference of 4.7 kcal/mol between the rate-limiting step and the isomerization barrier between the S-ylide and the W-ylide was detected, determining an equilibrium between both conformers. Consequently, *endo*-2,5-trans and *endo*-2,5-cis are both obtained. 

In the case of 2-formylpyridine ylides, the barriers of the S-ylide formation, that is the rate-limiting step, and S-ylide isomerization to W-ylide are competitive with a difference of only 1.4 kcal/mol. Consequently, the isomerization is negligible, and the obtained products (*endo*-2,5-trans and *exo*-2,5-trans) are both derived from S-ylide.

**Scheme 26:**
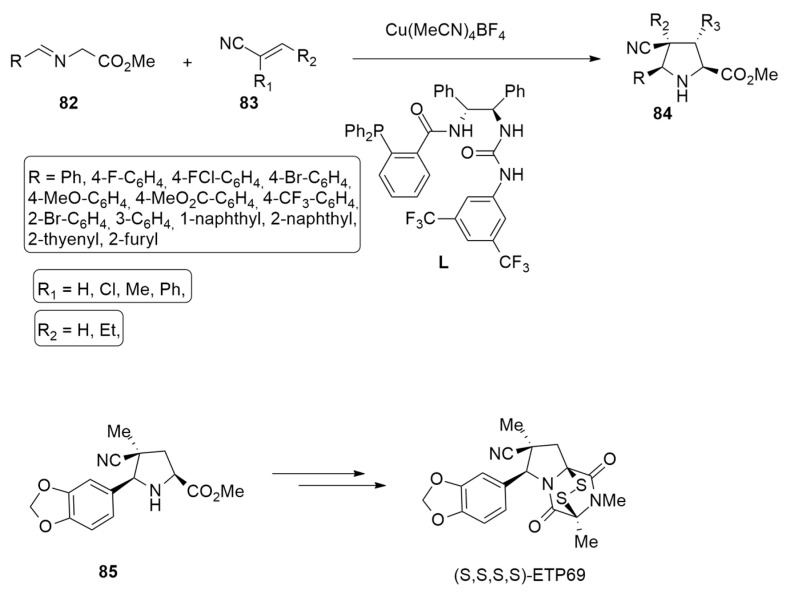
Synthesis of antitumor compound (*S,S,S,S*)-ETP69.

The enantioselective 1,3-dipolar cycloaddition of azomethine ylide **83**, produced by iminoester **82**, with acrylonitriles or methacrylonitrile **83** in the presence of Cu(CH_3_CN)_4_BF_4_ as a catalyst and the chiral phosphine−urea bifunctional ligand (**L**) has been achieved, leading to a series of highly substituted chiral cyano pyrrolidines **84** in high yields and with excellent diastereo- and enantioslectivity (up to 99:1 d.r., 99% ee) (Scheme 26). Using this protocol, the antitumor (*S,S,S,S*)-ETP69 was successfully synthesized with a 38% yield and with an enantiomeric purity greater than 99% using as the key compound the pyrrolidine **85** [45].

In order to investigate the enantio- and diastereoselectivity of this asymmetric cycloaddition, calculations were performed using DFT methods at the M11/6–311+G(d,p) level and the SDD basis set for Cu atoms. The solvent effects were determined through single-point calculations with the SMD solvation model, considering diethyl ether as a solvent.

The free energy profiles for an asymmetric 1,3-dipolar cycloaddition of azomethine ylides using the synthesized phosphine–urea bifunctional ligand **L** are considered.

**Figure 10:**
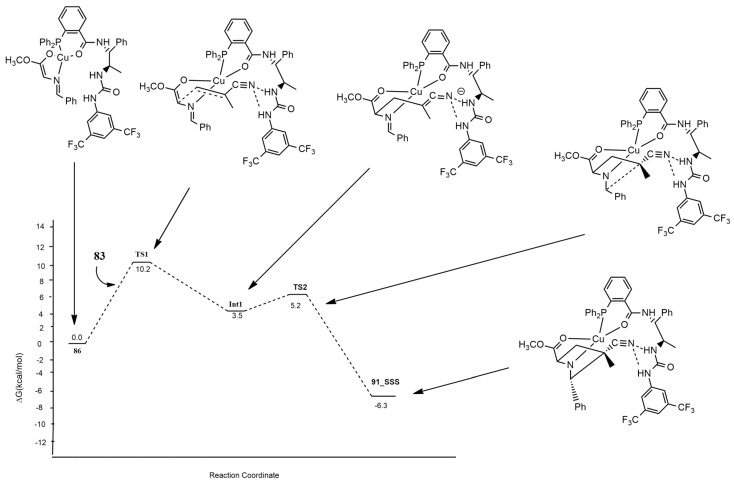
Energy profile leading to 91-SSS.

Firstly, iminoester coordinates to Cu, giving **86**. At this point, there is a nucleophilic addition of the *α*-carbon atom of the iminoester to the terminal carbon of acrylonitrile **83**. The subsequent cycloaddition rapidly gives the **91**-*SSS*. The nucleophilic addition is the enantioselectivity-determining step. Diastereoselectivity is determined by the lower barrier, leading to **91**-*SSS* with respect to the other stereoisomers. In fact, the corresponding transition state is stabilized by the formation of two different hydrogen bonds of the cyano group with the urea moiety (Figure 10). The authors finally concluded that both metal catalysis and organocatalysis are important for this reaction. Moreover, calculations reveal that the distortion energy has a determinant role in the enantioselectivity trend because of the steric effect between the phosphine ligand and the dipole.

**Scheme 27:**
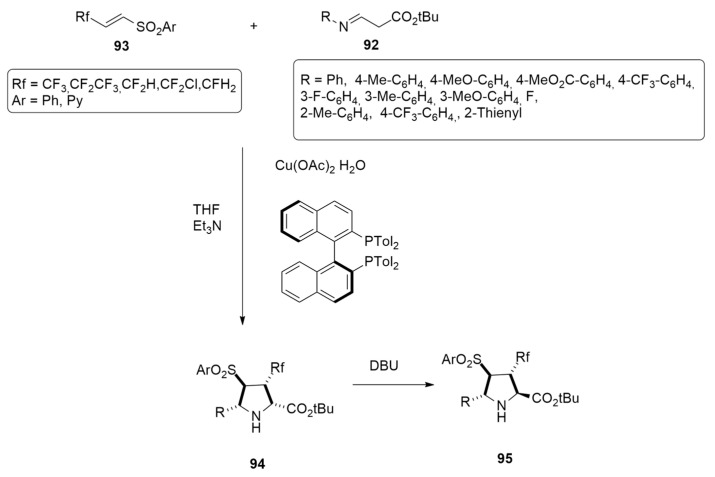
Synthesis of tetrasubstituted pyrrolidine carboxylates **94**.

The asymmetric 1,3-dipolar cycloaddition using glycine imino ester **92**, beta-fluoroalkyl alkenyl arylsulfones (-CF_3_, -CF_2_CF_3_, -CHF_2_, -CF_2_Cl, and CH_2_F) **93** and the (*S*)-tol-BINAP-Cu(II) complex, has been accomplished to synthesize the tetrasubstituted pyrrolidine carboxylates **94** with the simultaneous creation of four adjacent stereogenic centers [46] (Scheme 27).

**Figure 11:**
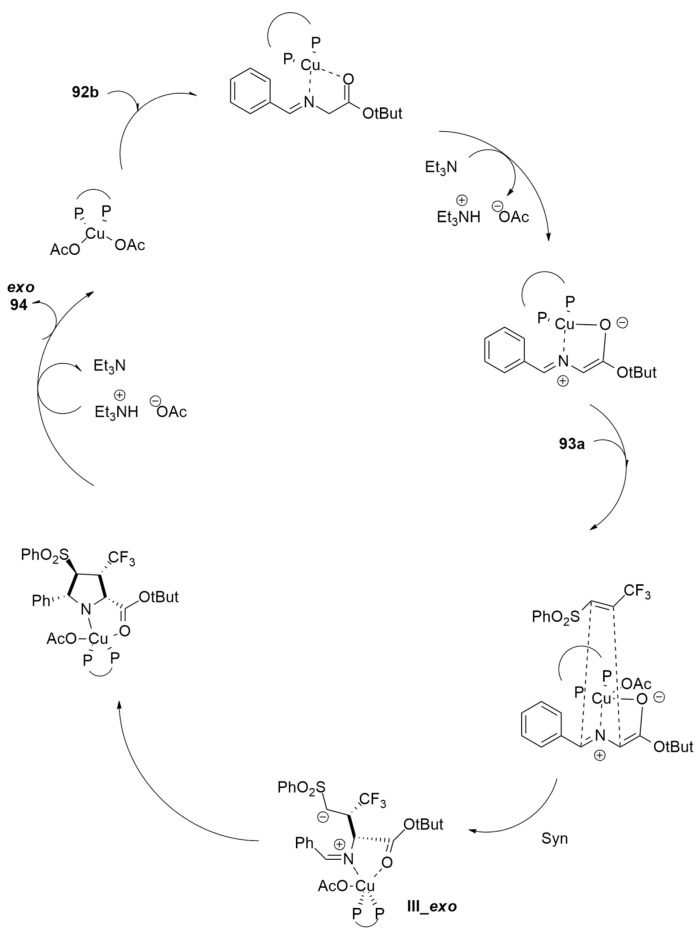
Catalytic cycle leading to compound *exo*-**94**.

The preferred diastereo- and enantioselectivity were rationalized through calculations at the M06-L/def2-TZVPP level. The mechanistic course of the reaction shown in Figure 11 is characterized by a stepwise nucleophilic addition in a head–head and/or tail–tail manner.

Firstly, the coordination of **92b** to **the** (S)-tol-BINAP-CuII complex allows deprotonation, producing the azomethine ylide **I**. The subsequent enantioselective Michael addition to the C=C double bond of the imonoester formed the zwitterionic intermediate **III**-*exo*. Finally, an intramolecular Mannich-type addition of **III**-*exo* to the iminium moiety on the Si-face and a proton transfer give compound *exo*-**94**. 

**Scheme 28:**
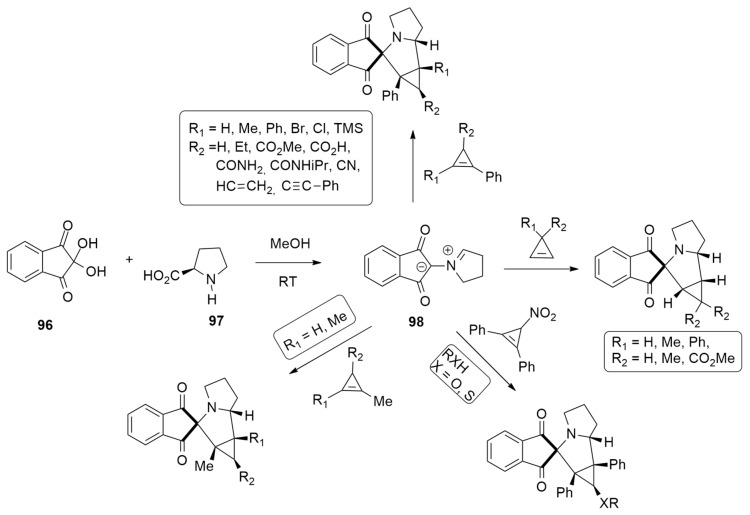
Stable azomethine ylide **98** from ninhydrin **96** and L-prolin **97**.

A stereo- and regioselective synthesis of cyclopropa[a]pyrrolizine derivatives, from moderate to excellent yields (33–95%), has been accomplished by Filatov et al. according to the reaction of azomethine ylide **98**, obtained from ninhydrin **96** and L-Prolin **97**, with different 1,2 substituted 3,3-disubstituted and 1,2,3-trisubstitued cycloprepenes (Scheme 28) [47]. Global electrophilicity indexes, the natural bond orbital (NBO) charges, FMO coefficients, Fukui function and free-energy profiles have been used to explain the 1,5-regio- and the *endo*- stereoselectivity of the cycloaddition process. 

**Figure 12:**
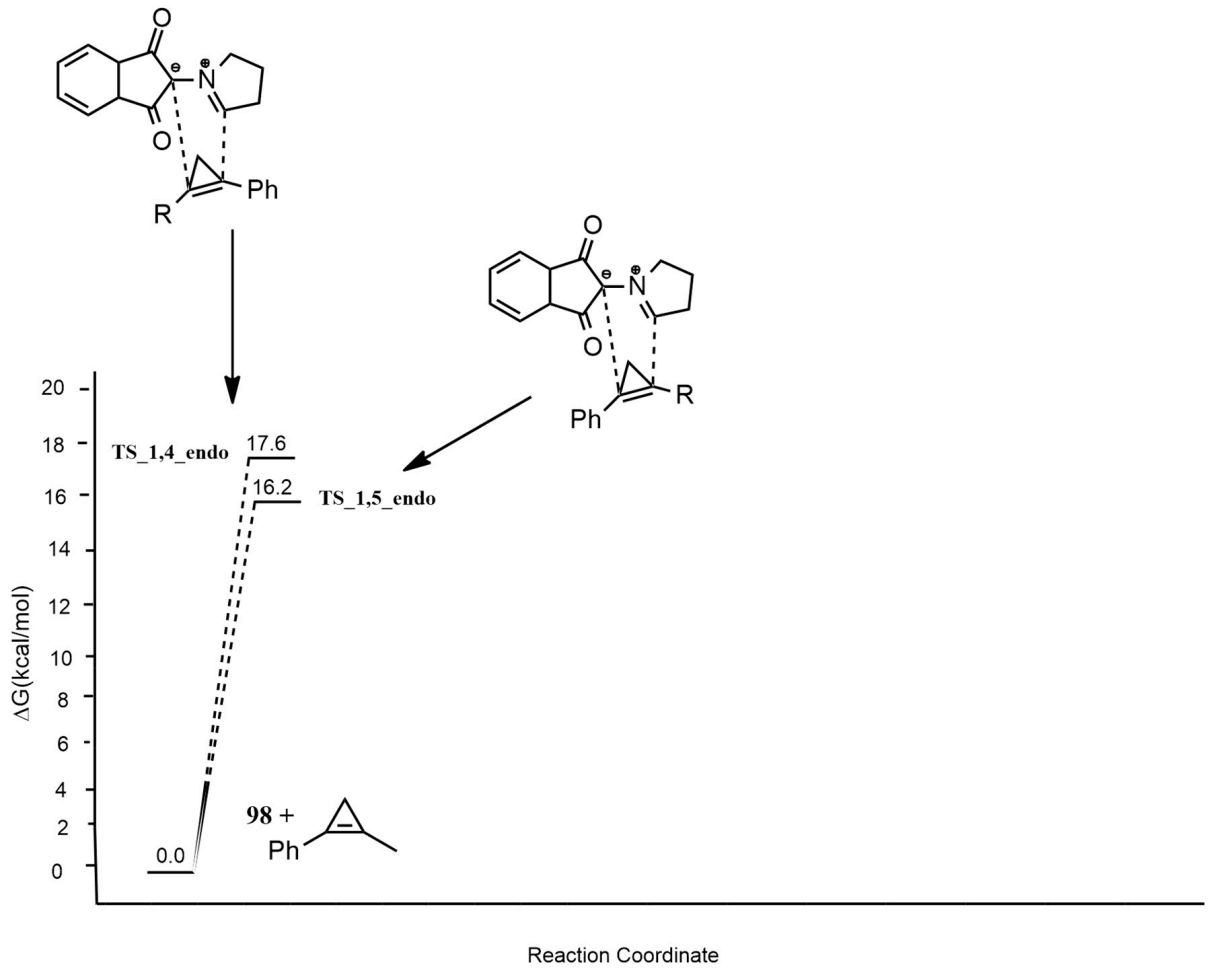
Free energy barriers leading to possible 1,4-*endo* and 1,5-*endo* compounds.

Optimizations were carried out with DFT/HF methods using the M11 hybrid exchange-correlation functional and cc-pVDZ basis set. Solvent (THF) was taken into consideration with the polarizable continuum model (PCM). The regioselectivity was investigated considering the two possible 1,4-*endo* and 1,5-*endo* routes. The cycloaddition has a one-step mechanism, and the 1,5-pathway is kinetically favored, leading to the 1,5-regioisomers as preferred (Figure 12).

The dipolaroid-like nature of the TSs suggests that this regioselective process is asynchronous and controlled by azomethine ylide **98**. Finally, the authors rationalize the stereoselection of the cycloaddition, considering both the *endo* and *exo* route and showing the lower barriers of the *endo*-via. The *endo* TSs are stabilized by an interaction between the N atom of **98** and syn-H atom of Csp^3^ of cyclopropene. In fact, the *s*-orbital in the HOMO of cyclopropene interacts with the nitrogen π-orbital of the ylide LUMO.

**Scheme 29:**
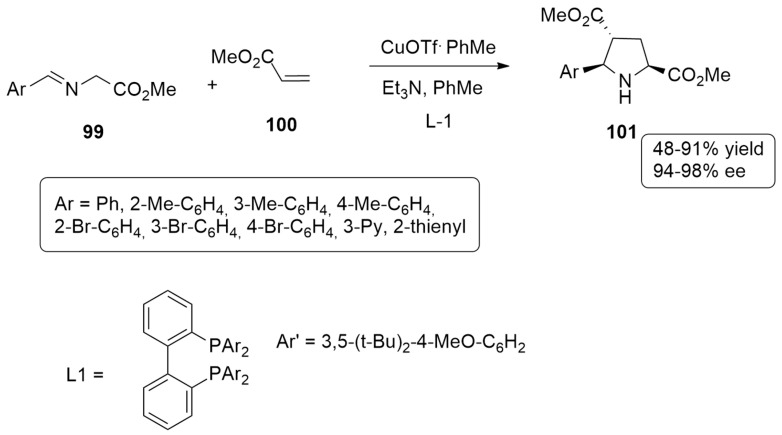
Enantioselective 1,3-dipolar cycloaddition using L1-CuOTf·PhMe complex and leading to *exo* adducts.

Caleffi et al. [48] have found that the L1-CuOTf·PhMe complex is able to efficiently promote an enantioselective 1,3-dipolar cycloaddition between imino esters **99** and electron-deficient alkenes **100**, leading to *exo* adducts **101** as major compounds (Scheme 29). 

**Scheme 30:**
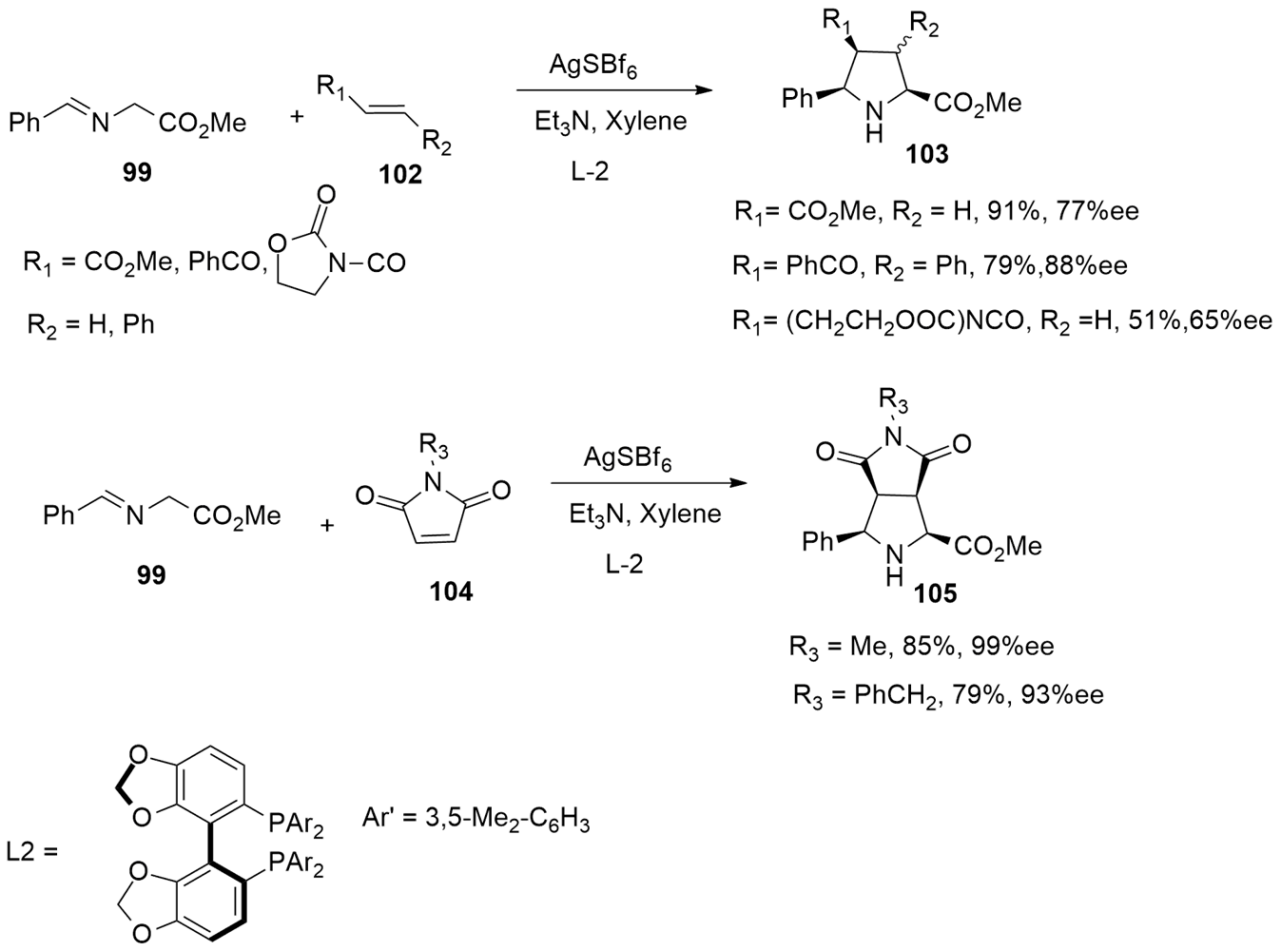
Enantioselective 1,3-dipolar cycloaddition using L2- AgSbF6-complex and leading to *endo* adducts.

On the contrary, employing L2-AgSbF6-complex,a, a diastereo-divergent process occurs with the formation of *endo*-cycloadducts as major cycloadducts (Scheme 30).

These different results have been explained with the aim of DFT calculations at the B3LYP/6-31G(d) level and PCM method with toluene as the solvent. 

The possible Cu(I) and Ag(I) complexes were studied. Because of the chelating character of the **L1** and **L2** chiral ligands, Cu and Ag atoms have a distorted tetrahedral coordination with the P atoms of the ligand and the N and O atoms of the iminoester. When dipolarophile attends the dipole, in the case of **INT1**, the coordination does not change. However, **INT1′** presents the replacement of one of the metal-P bonding interactions with a metal–oxygen interaction.

**Figure 13:**
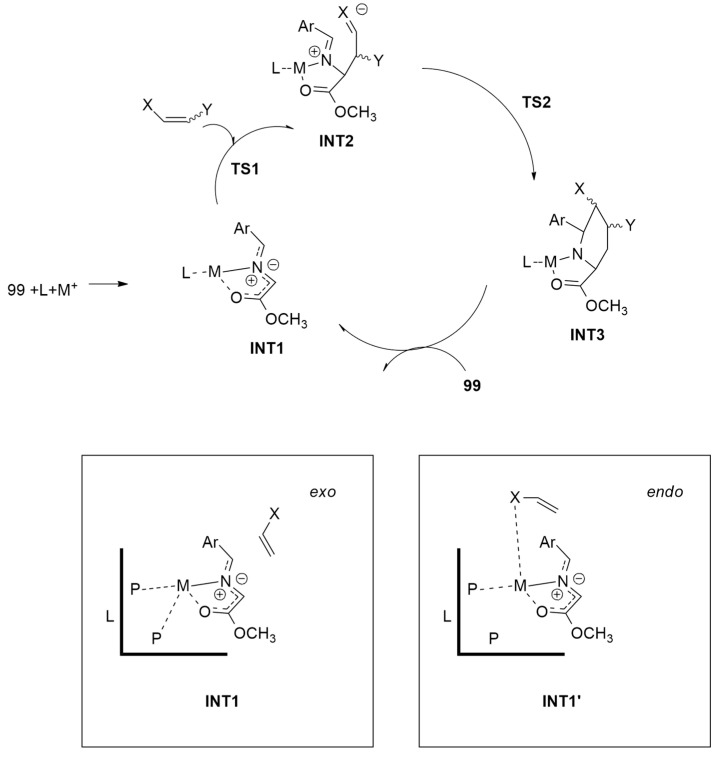
Catalytic cycle involving Cu(I) and Ag(I) complexes (A:M = Cu, X = CO_2_Me; B:M = Cu, X = CONMe_2_; C:M = Ag, X = CN; D:M = Ag, X = CO_2_Me).

Considering **INT1**, the coordination sphere of the metal is complete. Thus, the distal disposition between the cyano or carboxamido group with respect to the ester of the dipole determined the *exo* selectivity. On the contrary, the formation of **INT1′** and metal-O/metal-N bonding interaction is the driving force toward the obtainment of *endo*-products (Figure 13). 

**Scheme 31:**
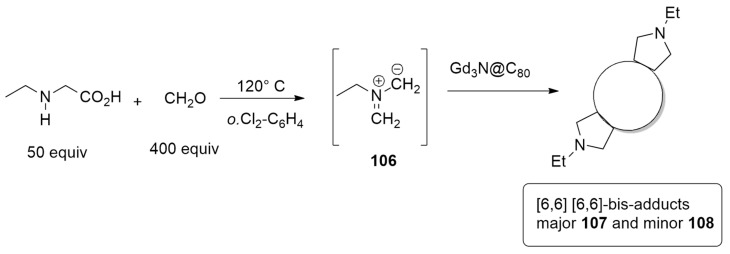
Synthesis of ethyl pyrrolidino adducts **107** and **108** using Gd3N@Ih-C80.

The reaction of the azomethine ylide **106** produced in situ by N-ethylglycine (50 equiv) and paraformaldehyde (400 equiv) at 120 °C for 15 min with Gd3N@Ih-C80 (Scheme 31) furnishes two ethyl pyrrolidino adducts **107** and **108** in a regioselective manner [49]. 

**Figure 14:**
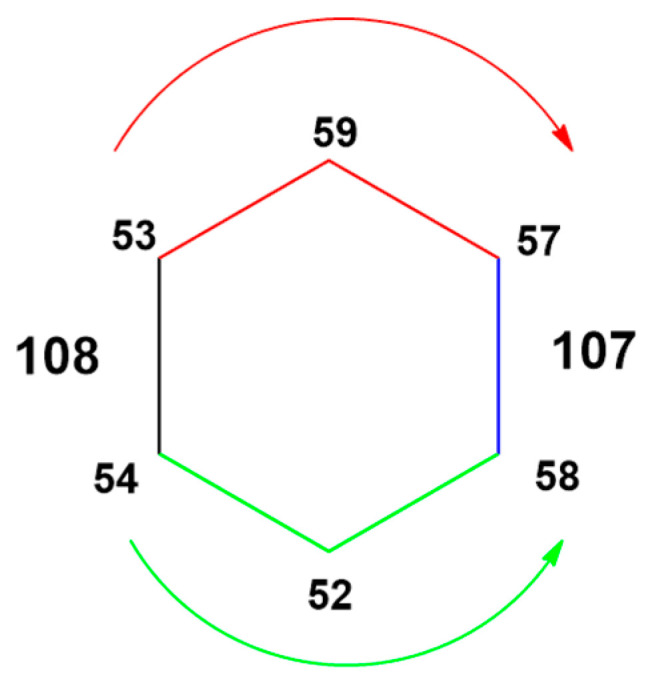
Isomerization pathway from minor **108** to major **107**.

The authors tried to elucidate the structures of the two cycloadducts using different techniques. X-ray diffraction study allowed for understanding that minor-bis-**108** has a C2-symmetric [6,6][6,6]-geometry on bond **53**–**54**. It is noteworthy that the Gd3N cluster is strictly planar and much less strained with respect to pristine Gd3N@C80, which presents the N atom out of the plane. The evaluation of the stability of adduct minor-bis-**2** under thermal conditions highlighted its isomerization to kinetic major-bis-1, perhaps through rearrangement. The inverse thermal conversion (from **107** to **108**) was not observed. The structure of **107** was determined through DFT calculations and optimizations at the BP86-D2/TZP level of theory. The major-bis-adduct-**107** has an asymmetric [6,6][6,6]-geometry with a second addition site on bond **57**–**58**. The trimetallic nitride template moiety is planar only in minor-**108**, while in the other case, the pyramidalization was detected. Finally, the isomerization from minor-**108** to major-**107** was studied. Calculations revealed that the reaction proceeds through a thermodynamically favored way that involves the formation of the [6,6][6,6]-bis-adduct on **54**–**52** (Figure 14). Therefore, the isomerization observed in this study is a [6,6] to [6,6] process. 

**Scheme 32:**
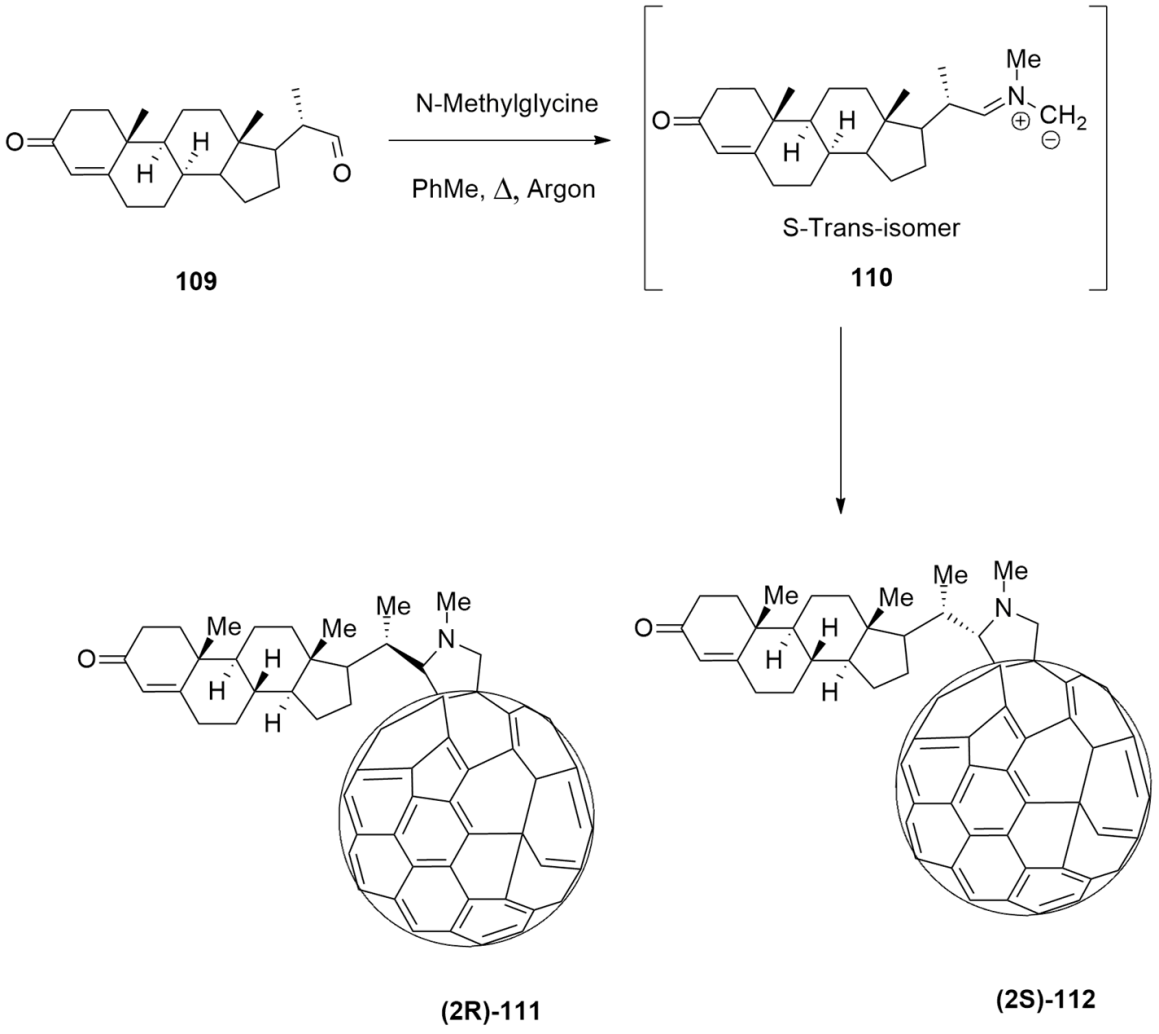
Reaction conditions: C60, N-methylglycine, toluene, reflux, Ar.

Fulleropyrrolidines *2R* and *2S* containing a stereocenter at carbon 2 of pyrrolidine ring, as potential HIV-1 protease inhibitors, have been, recently, synthesized by Alonso et al. [50]. These compounds have been accomplished in a diastereoselective fashion (ratio **111**:**112** 5:1) by the 1,3-dipolar cycloaddition reaction of the azomethine ylide **110**, produced by the enantiopure formyl steroid **109** and N-methylglycine, to [60]fullerene, according to Prato conditions (Scheme 32). 

**Figure 15:**
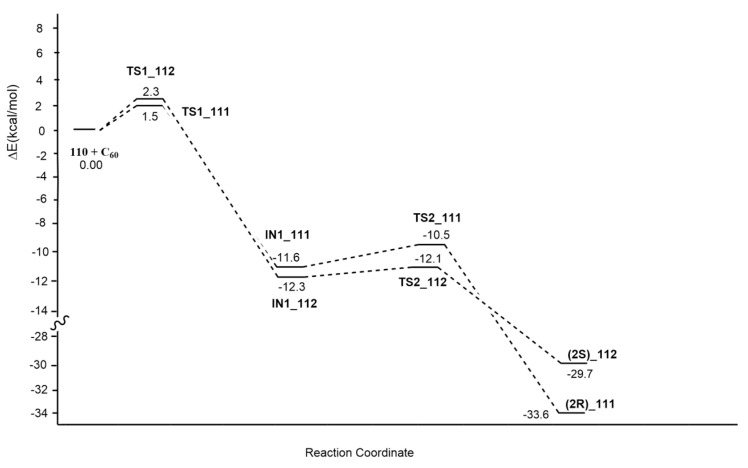
Energy profile of the 1,3-dipolar cycloaddition.

Firstly, using a combination of semiempirical and DFT methods, a conformational study was performed. In particular, the 1,3-dipole preferred the S-trans conformation. The evaluation of the nucleophilic character of reacting carbons of the dipole excludes that it influences the diastereoisomeric ratio. Optimizations at the B3LYP/6-31+G(d,p)/C-PCM = toluene//OLYP/6-31G(d):PM6 level allowed locating TSs, leading to the final cycloadducts. A two-step mechanism was detected with the first one established as rate-determining (Figure 15).

The computed ratio is in agreement with the experimental one (76:24 vs. 68:13). In **TS1_112**, the dipole is distorted with respect to the initial geometry in comparison with **TS1_111**. This effect is due to the steric hindrance determined by the methyl at C20, which influences diastereoselection. In conclusion, the attack of [60]fullerene on the *Re* face of the 1,3-dipole in the *S*-trans conformation is responsible for the final regioselectivity. Moreover, compound **111** is also thermodynamically favored.

**Scheme 33:**
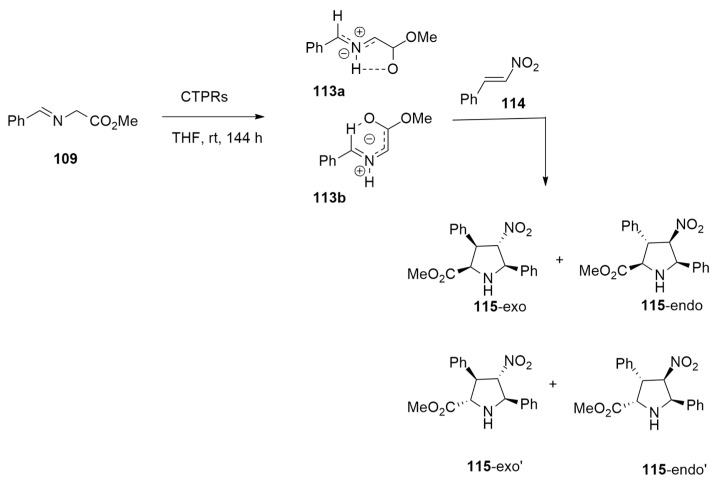
Synthesis of nitroproline esters using consensus tetratricopeptide repeat proteins (CTPRs) as biocatalysts.

Following a previous paper [51], Rivilla et al. have designed and synthesized consensus tetratricopeptide repeat proteins (CTPRs) as biocatalysts and used them to form nitroproline esters [52]. This process involves the formation of the azomethine ylide intermediate **113**, obtained from starting N-Benzylideneglycinate via enolization, promoted by CTPRs, and a subsequent reaction with nitrostyrene **114**. In particular, wild-type **CTPR1a** and **CTPR3a** catalyze the formation of the four pyrrolidine stereoisomers **115**–**116** (Scheme 33) with 40% yield in an equimolar ratio, while **CTPR1_wt** and **CTPR3_wt** catalyze the formation of only **115**-*exo* and **115**-*endo* with 20% yield in a 1:1 ratio. The formation of the four pyrrolidine isomers is rationalized to the in situ formation of azomethine ylides **113a** and **113b**. 

Starting imine **109** has two possible conformations in equilibrium through the rotation around the N−Cα bond: i.e., the homo-s-trans and homo-s-cis geometries that give dipoles **113a** or **113b**, respectively. This occurs through the proton transfer/abstraction of one Cα-H bond due to acid/base catalysis involving independent or connected active sites of CTPR proteins.

The mechanism leading to *endo*- and *exo*-**115a** adducts is concerted but asynchronous, while *endo’* and *exo’* were derived from a double suprafacial reaction. Also, the different stability and flexibility between CTPR and CTPRa series proteins can influence the course of the reaction. 

The authors performed QM/MM calculations, using **CTPR1a** and the ONIOM method at the M06-2X/6-311+G(2d,2p):dreiding//B3LYP/6-31G-(d,p):dreiding level. The high level includes imine **109**, nitroalkene **114**, and five residues (E2, K13, Y23, Y24, Q25), while the rest of the protein is treated at low level of calculations.

**Figure 16:**
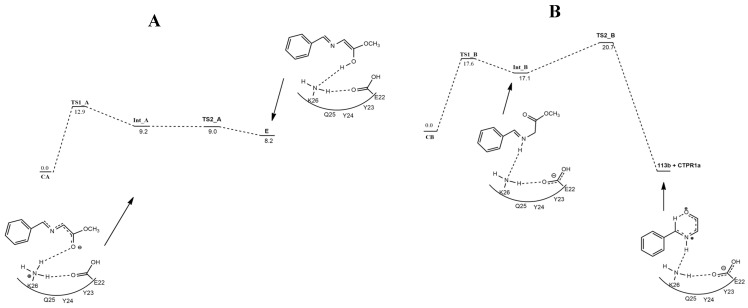
Energy profile leading to **113a** (**A**) and **113b** (**B**) starting from imine 1a in the homo-s-trans geometry and near the dyad E22/ K26 of CTPR1a.

Firstly, imine (homo-s-trans) and the dyad (E22/K26) form a complex **Ca** showing a H-bond involving the charged amino group of K26 and the carboxylate of E22 (Figure 16). Moreover, in **109**, the carbonyl O atoms coordinated to the protonated N atom of the amino moiety of K26, and a H atom of the methylene weakly interacts with the carboxylate moiety of the E22. Passing **TS1A** (about 13 kcal/mol), **109** is converted into enolate (**IntA**). Easily, without any significant barrier, enol **E** is obtained. From **E**, a prototropy reaction furnishes **113a**. The second way (**B**), leading to **113b**, involved **109** in the homo-s-cis geometry that, interacting with **CTPR1a**, gives the complex **Cb**, which is less stable than **Ca** by 4.4 kcal/mol. Starting from **Cb**, iminium cation **INTB** is obtained by the protonation of **109** by an ammonium group of K26. This step is endoergoic. Finally, passing **TS2B**, azomethine ylide **113b** is formed.

The two reaction profiles are almost isoenergetic, and so the formation of the two possible azomethine ylides **113a** and **113b** is competitive.

**Figure 17:**
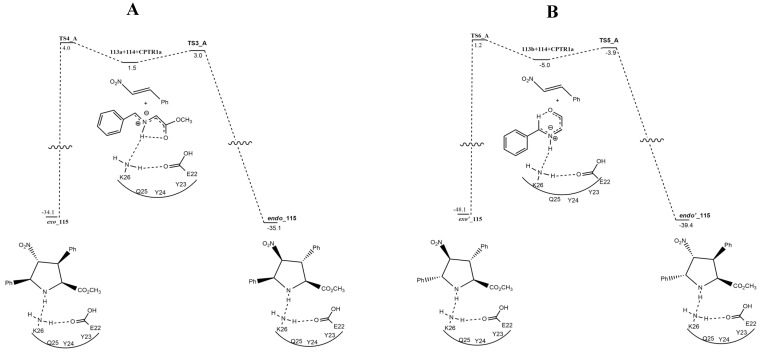
Energy profile of the formation of adducts *endo/exo*-**115** (**A**) and *endo′/exo′*-**115** (**B**) starting from **113a** and **113b**, respectively.

Finally, the cycloaddition between **115a** and **b** with dipolarophile **114** is considered (Figure 17). Starting both from **113a** or **113b**, the formations of *endo* or *exo* compounds are in competition (**TS3** vs. **TS4** = 3.5 vs. 6.8 kcal/mol; **TS5** vs. **TS6** = 5.2 vs. 10.3 kcal/mol) and so a mixture of cycloadducts is obtained, although the *endo* products are favored. The reinitiating of the cycle is guaranteed by the recovery of the catalytic couple E22/K26. This is allowed by the weakening, in the cycloadducts, of two interactions (i.e., the one between the NH group of the cycloadduct and the N atom of the K26 residue, and the H bond present in turn between the N atom of K26 and the neutral carboxy group of E22). 

**Scheme 34:**
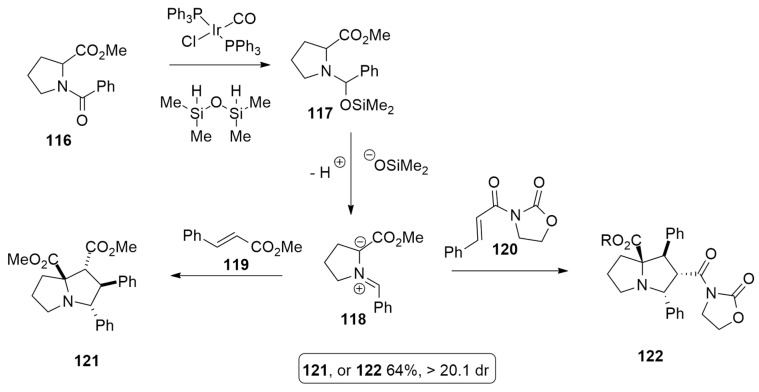
Synthesis of compounds **121** and **122**.

Yamazaki et al. [53] have recently reported the synthesis of various substituted pyrrolidines, in regio- and diastereoselective fashion, by the 1,3-dipolar cycloaddition of azomethine ylides, under mild condition, using iridium complex as the catalyst. The synthesis occurs in one step, involving N-benzoylprolinemethylester **116**, chlorodicarbonylbis(triphenylphosphine)iridium(I) complex and 1,1,3,3-tetramethyldisiloxane. As described in Scheme 34, the azomethine ylides **118** involved in the process is obtained through a partial reduction of the amide group by Vaska’s complex and tetramethyldisiloxane, which is followed by the silanoate elimination of **117**. Finally, the dipole reacts with methyl cinnamate **119** or N-enoyl oxazolidinone **120** as dipolarophiles to afford the corresponding pyrrolidine derivatives **121** and **122**, respectively, in 64% yields. 

In order to clarify the different selectivity observed for the two reactions, DFT calculations were performed at the BP86/TZ2P level.

**Figure 18:**
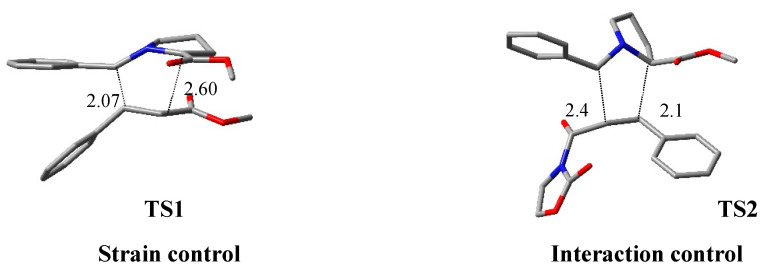
Three-dimensional (3D) plots of the preferred TSs when dipolarophile is methyl cinnamate (**TS1**) or N-enoyl oxazolidinone (**TS2**).

The diastereoselectivity is influenced by strain factors or interaction energy in the case of methyl cinnamate **119** or oxazolidinone **120**, respectively. In both reactions, four **TSs** can be located, but the lower barrier was shown to be different: **TS1** for methyl cinnamate and **TS2** for N-enoyl oxazolidinone (**TS2**) (Figure 18). Using the activation strain model (ASM), the ΔE^≠^ was distinguished into two contributions: the strain energy or ΔE^≠^strain, determined by deformation of reactants, and the interaction energy (ΔE^≠^int) between deformed reagents. When methyl cinnamate is the dipolarophile, the selectivity of the favorite **TS** is ruled by ΔE^≠^strain, while in the **TS** preferred for N-enoyl oxazolidinone is ΔE^≠^int.

The same author, in a previous paper [54], correlated the degree of asynchronicity of Diels–Alder reactions with strain energy value. In general, an asynchronous TS, in which the pyramidalization of the C atoms is minimized, shows a less destabilizing E_strain_; this is the case of TS1. When an oxazolidinone is part of a dipolarophile, the E_int_ value prevails on E_strain_ as in **TS2**. Moreover, the molecular electrostatic potential analysis (MEP) highlighted that **TS2** has the forming C−C bond.

**Scheme 35:**
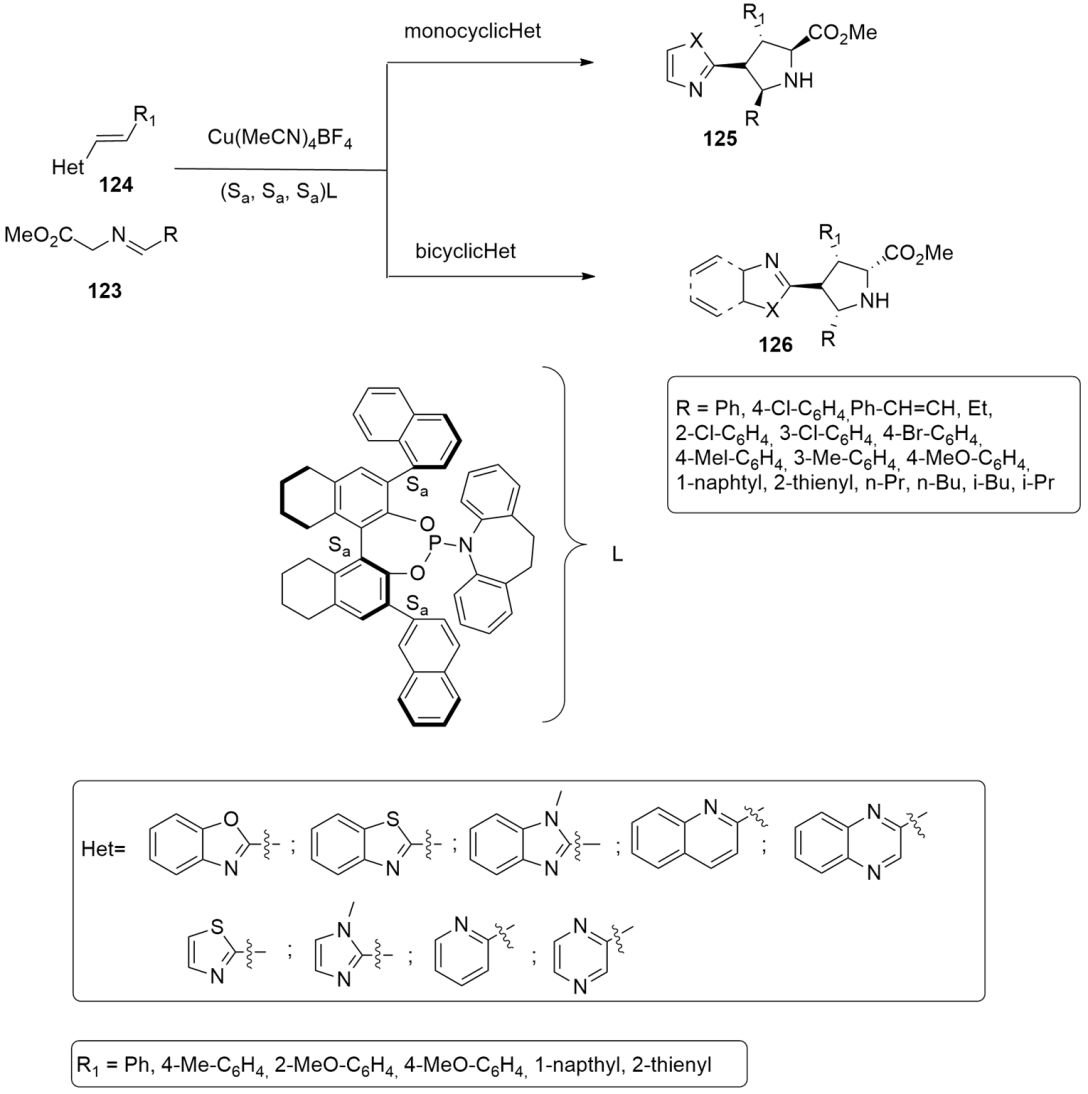
Synthesis of pyrrolidines **125** and **126**.

Cu(I) complexed with a monodentate, triple-homoaxial chiral, phosphoramidite ligand (L) was employed by Chang et al. [55] in the synthesis of enantioenriched pyrrolidines **125** and **126**, obtained by the 1,3-dipolar cycloaddition of azomethine ylides, produced by **123**, with heteroaryl alkenyl derivatives **124** not possessing strong electron-withdrawing substituents (Scheme 35). 

The exclusive diastereoselectivity and the excellent enantioselectivity are explained by the uncommon ability of the complex Cu(I)-L to activate both the dipole and the dipolarophile involved. 

**Figure 19:**
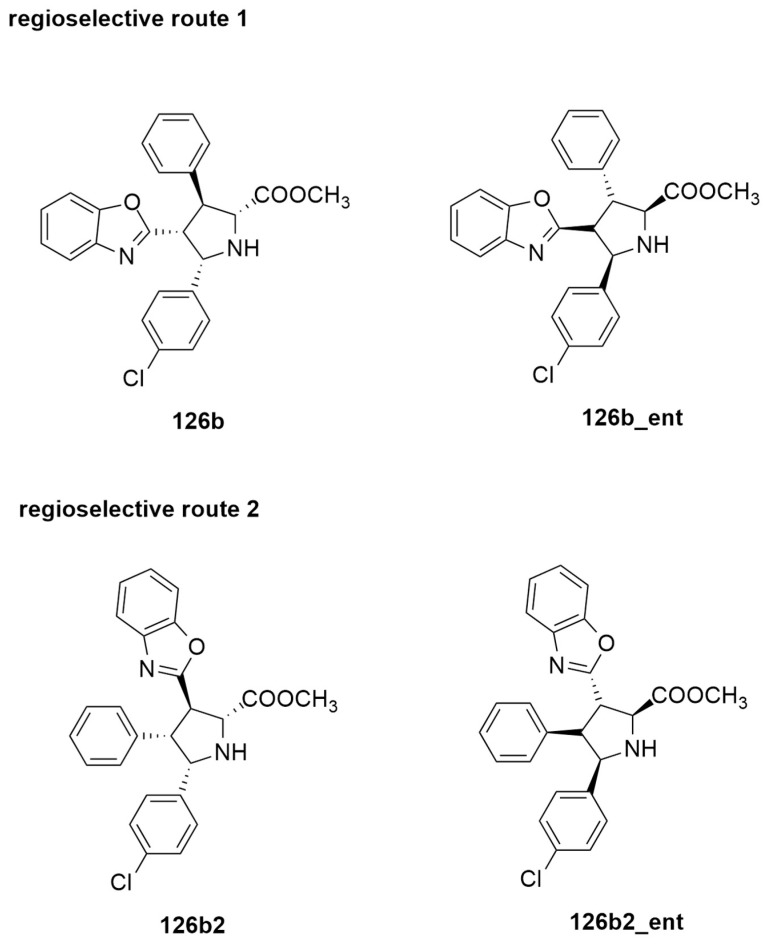
Regioisomeric routes for the synthesis of enantioenriched pyrrolidines **126**.

The stereochemical outcome of the reaction leading to **126b** (Figure 19) was clarified through DFT calculations using the M06-L method and the SDD basis set for Cu and 6-31G(d) for the other atoms. Two different regioselectivity routes are possible as reported in Figure 19.

The Cu(I)-L complex prefers the coordination mode in which the 4-Cl-C_6_H_4_ group does not suffer steric repulsion with the amido moiety.

**Figure 20:**
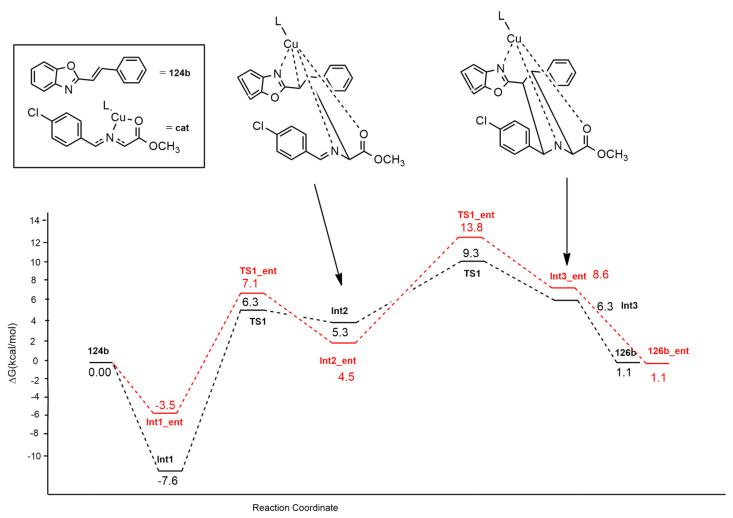
Energy profile of the reaction leading to **126b** (black dotted line) or **126b**_**ent** (red dotted line).

Mechanistic investigation highlighted that the reaction occurs in two steps. Passing a first barrier (**TS1**), a zwitterionic intermediate (**INT2**) is obtained with the formation of the C1−C3 bond between the ylide and the alkene. The second step regards the formation of a new C−C bond determining cyclization. The cycloaddition is the rate-determining step of the process (Figure 20). The **endo_126b**, obtained by the attack from the upper side of the ylide, is the main product. The formation of its enantiomer **126b_ent** is disfavored by the nonbonding interactions, present in **TS2**, between H4 of naphthol and benzo[d]oxazole moiety. All the other considered ways present higher barriers, and the formation of the other products is excluded. The completion of the catalytic cycle with the regeneration of L occurs thanks to the exchange between the product and another molecule of azomethine ylide. Computational data are in very good agreement with experimental results, showing that the proposed phosphoramidite ligand (rigid, sterically bulky and with a triple-axial chirality) is very suitable in providing a chiral environment around the cycloaddition.

**Scheme 36:**
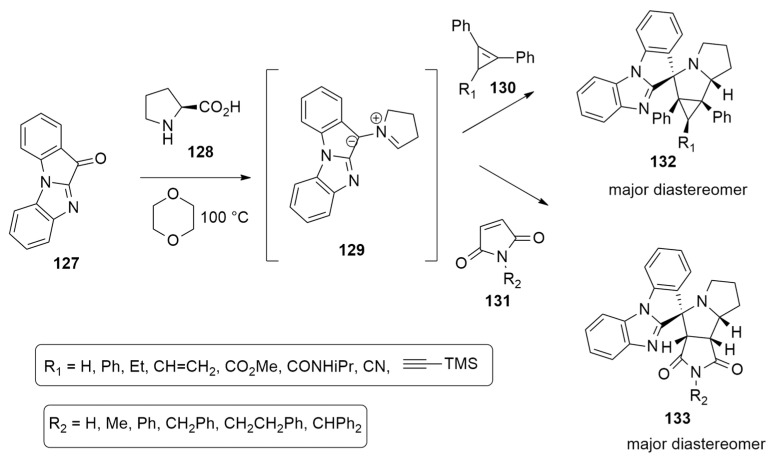
Synthesis of spiro heterocyclic compounds **132** and **133**.

The azomethine ylides **129**, obtained from the tetracyclic ketone 11H-benzo[4,5]imidazo[1,2-a]indol-11-one **127** and L-proline **128**, have been utilized by Filatov et al. [56] to synthesize several spiro-heterocyclic compounds when they react with cyclopropenes **130** or maleimide **131**, which are differently substituted (Scheme 36). 

With the support of DFT calculations, performed at the M11/cc-pVDZ level using the PCM method to treat the solvent (1,4-dioxane), the mechanism was investigated, considering the azomethine ylide **129** that reacts with 1,2-diphenyl-3-vinylcyclopropene.

Compound **127** and L-proline **128** give azomethine ylide **129** in two possible conformations: S-shaped (**129_A**) and W-shaped (**129_B**). Their precursors are diastereomeric oxazolidin-5-ones, **INT1_A** and **INT1_B**, which are obtained by the condensation of **127** and **128**. Then, the 1,3-dipolar cycloreversion, leading to 1,3-dipoles (**129_A, 129_B**), occurs in two steps passing through *endo* TSs. The first one is the rate-determining step, and the barriers of the two routes highlighted that **AY-1** and **AY-2** are obtained in an equimolar ratio.

**Scheme 37:**
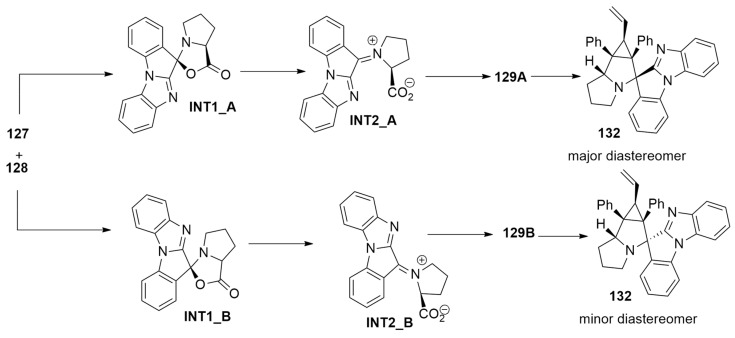
Mechanistic route leading to compound **132**.

The *endo* pathway, starting from ylide **129_A**, is preferred with respect to the *endo* route from **129_B** by about 0.9 kcal/mol. Cycloadduct **132** is thermodynamically favored, and it is present in greater quantities in the final mixture (Scheme 37).

**Scheme 38:**
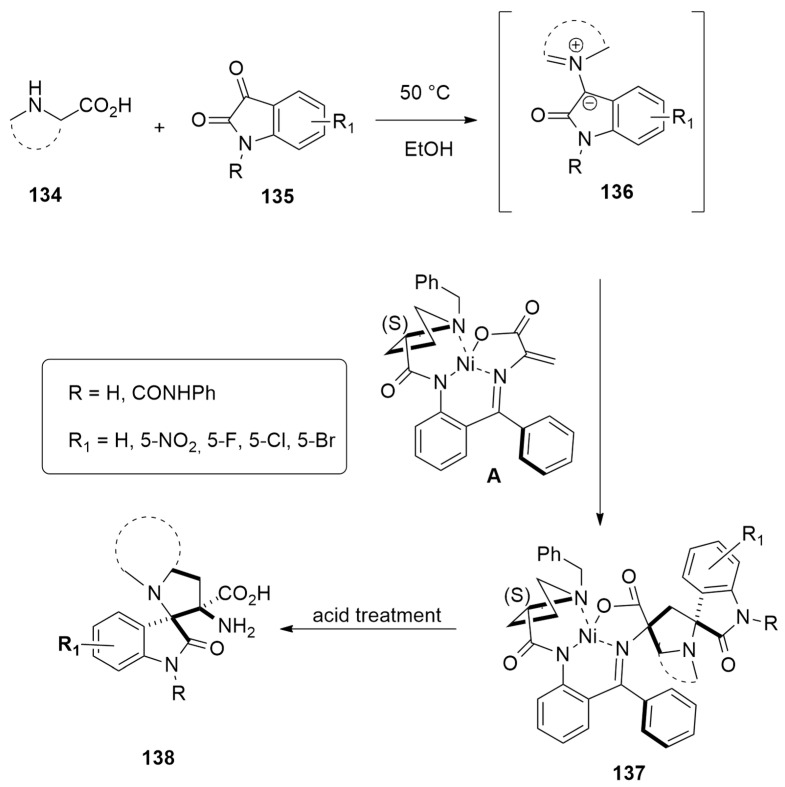
Reaction of amino acids **134** with isatins **135** and Belokon’s chiral Ni(II) complex leading to cycloadducts **138**.

Chiral unnatural amino acids **138** in enantiomeric pure form, characterized by the presence of 3-spiropyrrolidine oxindole skeletal, have been obtained in moderate to good yield (40–86%) and with high diasteroeselectivity (>20:1), by a three-component reaction, involving as a key intermediate the azomethine ylides **136** [57]. Thus, the reaction of various amino acids **134**, with different isatins **135** and Belokon’s chiral Ni(II) complex **A**, at 50 °C for 24 h in ethanol, furnishes after a usual work-up of the desired cycloadducts **138** via the initial cycloadducts **137** (Scheme 38).

The regioselectivity of the reaction involving (S)-proline and sarcosine was investigated through DFT calculations at PBE0-D3BJ/def2SVP/CPCM(EtOH) level. The selected functional (PBE0) proved to be accurate for studying the organic reactions. Considering the CN bond, the obtained azomethine ylide has two possible geometries: *Z* or *E*. The approach to the Ni complex can differently occur: (i) in a tail-to-tail (tt) way, directing the bulkier moieties toward each other, or in a tail-to-head manner (th) with the same groups in the opposite direction; (ii) attacking the alkene C atom of the complex from the top and bottom side; (iii) determining the configuration of C3 of isatin. 

The mechanistic investigation locates 16 different TSs for Pro and sarcosine.

**Figure 21:**
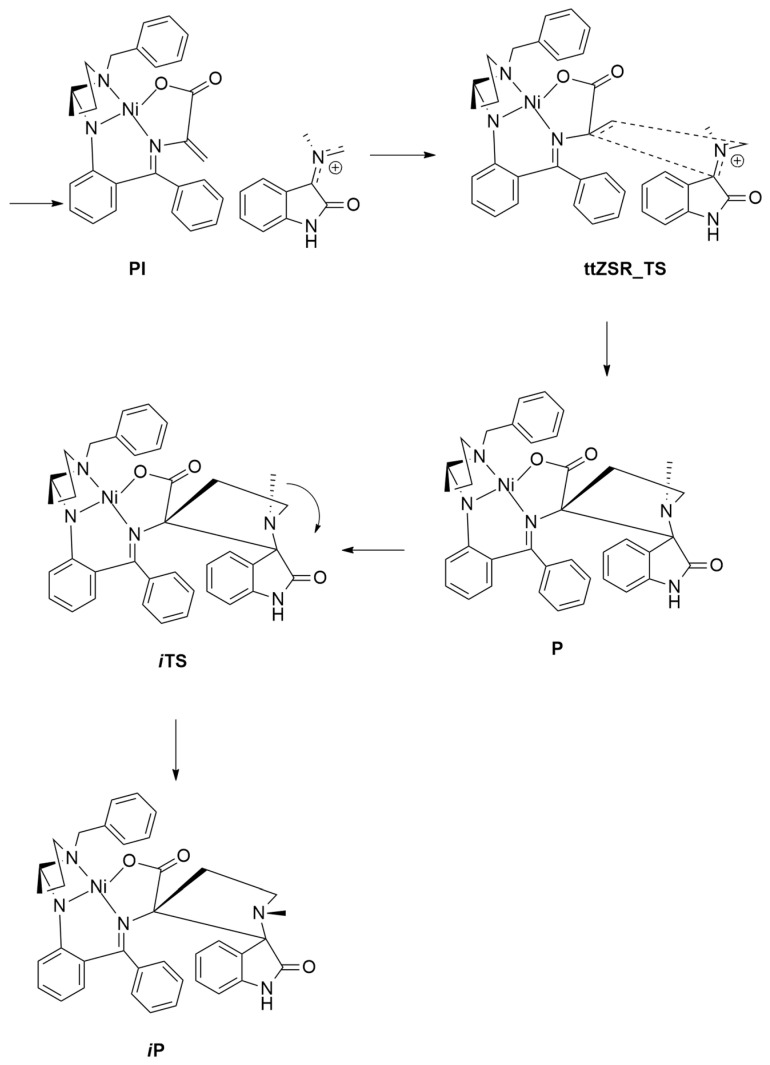
Located structures for the preferred way (*ttZSR)* involving sarcosine.

In detail, the (*S*)-1 Ni-complex forms with azomethine ylide a π-complex (**PI**) with the groups of the N atom of ylide with *endo* orientation (Figure 21). Starting from this complex, the pericyclic reaction occurs, passing a transition state (**TS**) characterized by a planar configuration of ylide. In both cases, *ttZSR***_TS**, leading to adduct (**P**) with ttZSR configuration, was shown to be preferred. Moreover, from the product, the N inversion is possible. Experimental results confirmed calculations for sarcosine, while when Pro is an aminoacid, a cycloadduct with the *thZSR* configuration is achieved. In the case of sarcosine, the reaction is under kinetic control and a ttZSR-adduct is formed. The retro-cycloaddition has a too high barrier to happen. Instead, in the case of proline, the retro-reaction is possible as experimentally verified. Calculations also showed that passing a barrier of 28.7 kcal/mol, the rearrangement from ttZSR to thZSR starting from product **iP** can occur at room temperature. The *thZSR*-**iP** adduct is thermodynamically preferred over the possible regioisomeric **P** and **iP** products. So, in this last case, the outcome of the reaction is influenced by both thermodynamic and kinetic control.

**Scheme 39:**
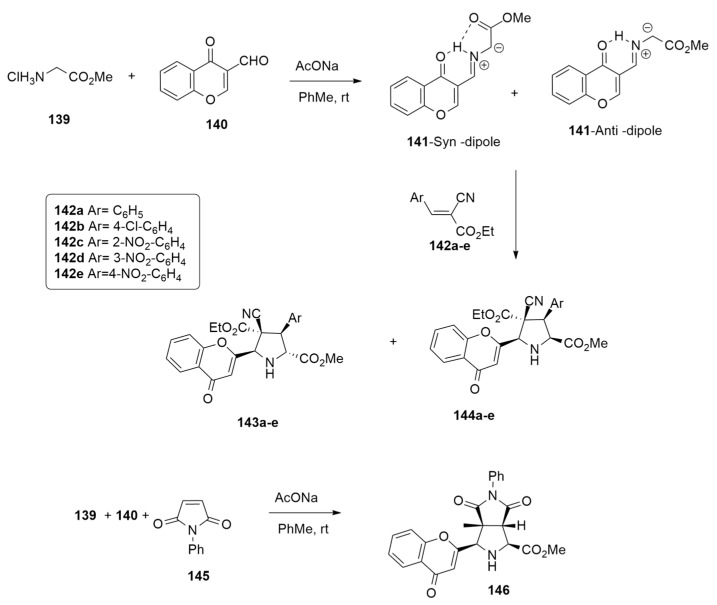
The 1,3-dipolar cycloaddition of azomethine ylide **141** with glycine ester **139** leading to *endo* adduct.

Radwan et al. [58] have studied the metal-free 1,3-dipolar cycloaddition of azomethine ylide **141**, derived from 3-formylchromone **140**, with glycine ester **139**, and arylidenes **142a**–**e** or phenylmaleimide **145** (NPM) in the presence of AcONa in toluene at room temperature. The cycloaddition process, in the case of arylidenes **142a**–**e** as dipolarophiles, leads to a mixture of *endo* (**143a**–**e**) and *exo’* (**144a**–**e**) adducts in excellent yield (92–88%) and with an *endo* product as the major diastereomer. On the contrary, using NPM **145** as the dipolarophile, the *endo* cycloadduct **146** was the only obtained product (86% yield) (Scheme 39).

**Scheme 40:**
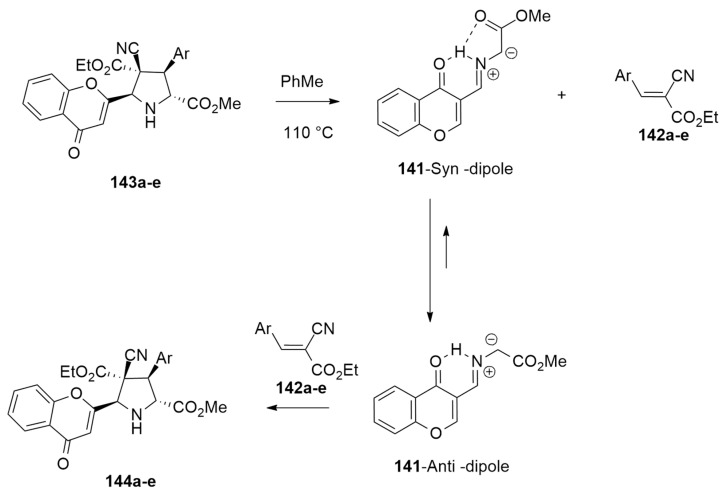
The 1,3-dipolar cycloaddition of azomethine ylide **141** with glycine ester **139** leading to *exo* adduct.

Interestingly, when the reaction of **139**, **140** and **142a**–**e** was conducted at reflux temperature (110 °C), only cycloadducts **144a**–**e** were obtained. These results can be easily rationalized considering that the kinetic cycloadducts **143a**–**e** at high temperature are transformed, via the in situ retro-1,3-dipolar cycloaddition and recyclization, in the thermodynamically *exo’* derivatives **144a**–**e** (Scheme 40).

The mechanism of the reaction was investigated using DFT calculations in order to clarify the role of AcO_2_H in this cycloaddition reaction. Optimizations were performed at the wB97xd/6-31G(d,p) level of theory.

The first studied step was the formation of the *syn*-/*anti*- dipoles, focusing attention on acetic acid.

**Figure 22:**
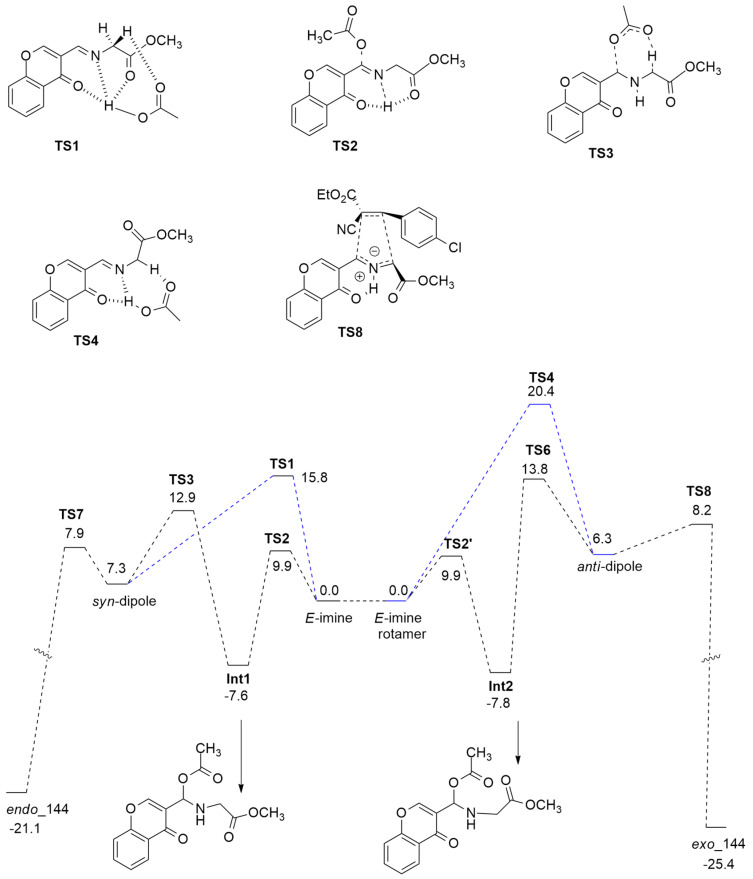
Energy profile of the 1,3-DC of azomethine ylide **141** with glycine ester **139** leading to *endo* and *exo* adducts.

In the first possible pathway, starting from (E)-imine (blue dotted lines in Figure 22), the protonation of the N atom, determined by AcOH, occurs in a concerted way with respect to the deprotonation of the α-hydrogen atom of the imine (**TS1**). Instead, the second possible route (black dotted lines) is characterized by a two-step mechanism with AcOH addition/elimination (**TS2** and **TS3**). 

The H-bonds in **TS1** allow a more stable trifurcated structure with the obtainment of the *syn*-dipole preferred from a kinetic point of view. Transition state **TS4**, characterized by strong H-bonds that give a bifurcated structure and lower steric hindrance, is the favorite way to achieve the *anti*-dipole.

Then, the mechanistic study was extended to the [3 + 2] cycloaddition with dipolarophile **142a**: starting from a dipole in *syn* or *anti* conformation, passing two low barriers **TS7** and **TS8**, and leading to *endo*- and *exo*’-cycloadducts **143a** and **144a**, respectively. These data are in agreement with experimental results that show the *endo*-adduct, kinetically favored, as the major product.

The barriers highlighted the possibility of retro-cycloaddition at room temperature starting from the endo **143a** followed by the isomerization of a *syn*-dipole into an *anti*-dipole. Instead, *exo*’-adduct **144a** is thermodynamically preferred. In general, IRC analysis showed for the process an asynchronous concerted mechanism.

**Scheme 41:**
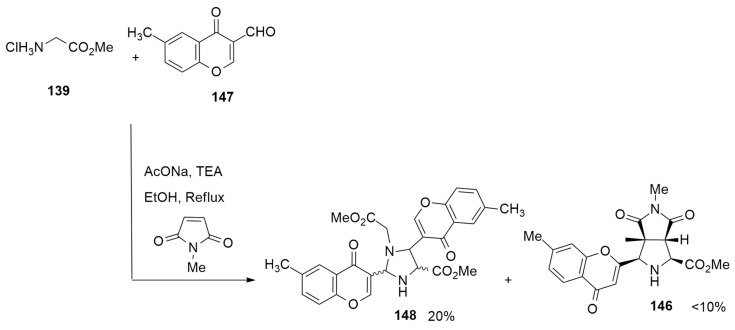
Sansano reaction [59].

Finally, the computational study proceeded considering the [3 + 2] cycloaddition of glycine imino ester **139** with NPM (*N*-phenylmaleimide) in the presence or not of the AgOAc catalyst. Calculations were carried out at the same level as above, and the Lanl2dz basis set was used for the Ag atom. This reaction is experimentally fast and, without metal, gives a reaction that is not reversible, leading to highly stable cycloadduct **146**. On the contrary, in the presence of Ag as the catalyst, a mixture of decomposition products is obtained (see Scheme 41).

The barriers for the formation of the *syn*-dipole and following the reaction with NPM without a metal catalyst are analogous to those above described. The preferred cycloadduct *endo*-**143a** is more stable, and so the reaction is fast and irreversible.

The introduction of the catalyst AgOAc shows low barriers leading to Ag-azomethine ylide (7.4 kcal/mol) and cycloadduct (4.9 kcal/mol). However, the complexation of Ag with the starting imine is more stable than the complexation of metal with a final cycloadduct, rationalizing the experimental obtainment of decomposition products.

#### 2.2.2. Nitrones

The cycloaddition of nitrones to alkenes represents a fast and elegant way to prepare isoxazolidines, which are very important compounds/intermediates in organic chemistry [60]. These cycloadducts have found wide applications as synthons in total synthesis by their conversion into 1,3-aminoalcohols and alkaloids. Furthermore, the isoxazolidine system is a mimetic of the ribose unit, and it has recently been exploited in the synthesis of modified nucleosides having antiviral and antitumoral activity [61,62].

**Scheme 42:**
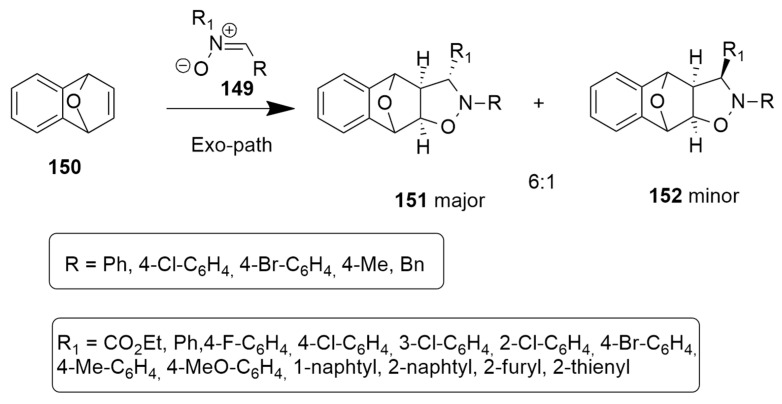
Synthesis of fused bicyclic tetrahydroisoxazoles.

Fused bicyclic tetrahydroisoxazoles, without the use of catalysts or additives, were synthesized by Yao et al. [63] by the 1,3-dipolar cycloaddition of nitrones **149** with oxa(aza)bicyclic alkenes **150** (Scheme 42). Final compounds were obtained, as a diastereoisomeric mixture, in very good yields (76–99%). This reaction is an important example of efficient [3 + 2] cycloaddition, conducted in mild conditions, to obtain highly functionalized compounds (Scheme 42).

**Scheme 43:**
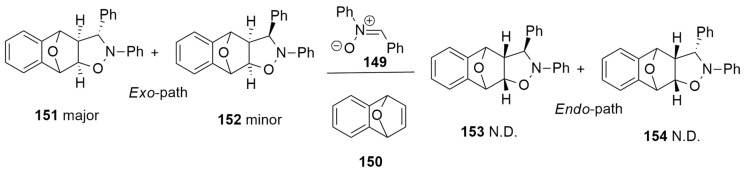
*Endo* and *exo* pathways of the 1,3-DC leading to fused bicyclic tetrahydroisoxazoles.

The mechanism of the reaction, reported in Scheme 43, was studied through optimizations at the B3LYP/6-311G(d,p) level and using the PCM solvation model and toluene as the solvent. 

The *endo* and *exo* routes, that give **153** and **154** and **151** and **152**, respectively, were taken into consideration. All the possible TSs were located. The *endo* pathway is disfavored, and the barriers are very close to 50 kcal/mol. Considering the *exo* pathway, with a lower barrier that is close to 30 kcal/mol, the obtainment of **151** is preferred over **152** by about 3 kcal/mol, which is in very good agreement with the experimental determined diastereoselectivity. 

**Scheme 44:**
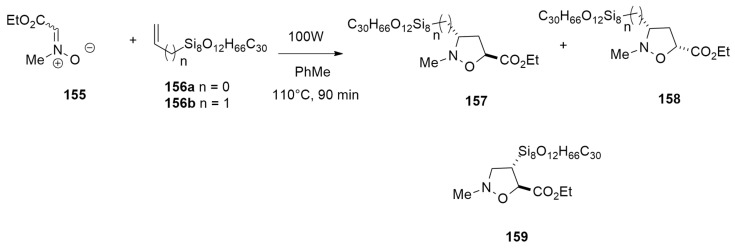
The 1,3-dipolar cycloaddition reactions of N-methyl-C-ethoxycarbonylnitrone **155** with allyl-heptaisobutyl-POSS **156**.

The 1,3-dipolar cycloaddition reactions of N-methyl-C-ethoxycarbonylnitrone **155** with allyl-heptaisobutyl-POSS **156b**, under microwave irradiation, produce two cycloadducts: **157a** and **158a** with a high control of regio- and stereoselectivity in a 5.7:1 ratio, respectively. Differently, the reaction of **155** with **156a** leads also to the formation of adduct **159** with inversion of the regioselectivity, although in moderate yield (10%), but high stereochemical control (only trans adduct) (Scheme 44) [64].

The experimental results, in particular regio- and stereoselectivity, were rationalized by DFT calculations at the B3LYP/6-31G(d) level, investigating the reaction of POSS-substituted olefin **156a,b** with nitrone **155**. The different TSs, deriving from **155** in the E or Z configuration, and the *exo* or *endo* approach of the dipolarophiles were considered with the POSS and -CO_2_Et moiety in cis or trans configuration. 

**Figure 23:**
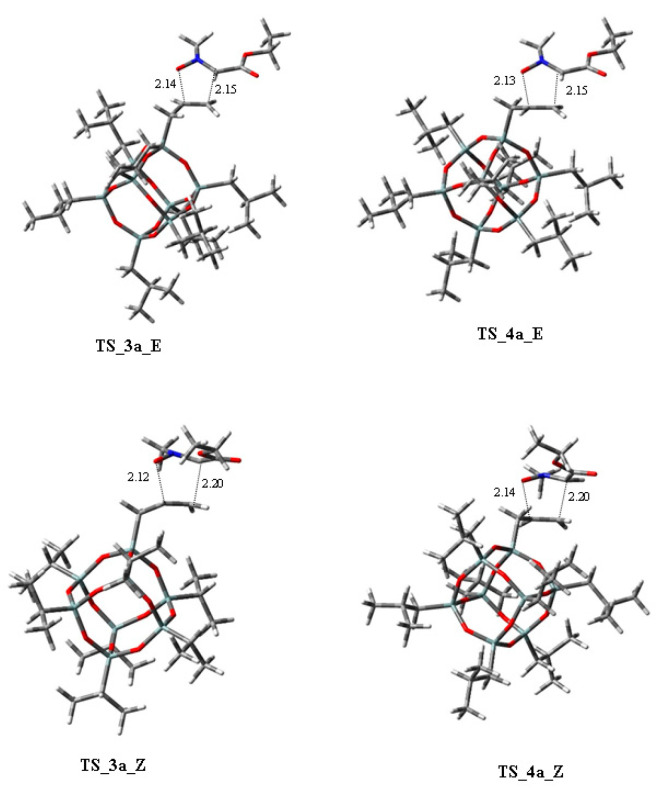
TSs of reaction between **155** and **156a** leading to stereoisomers **157a** and **158a**.

Figure 23 contains the 3D plots of the TSs leading to **157a** and **158a**. The favorite **TS 157a**_E/Z furnished trans adduct **157a** with 95% yield, while **TS 158a**_E/Z barriers are higher in energy and gave a 3.1% yield of cis derivative **158a**. The barrier relative to the formation of **159** is higher (>30 kcal/mol), and only 2% yield of this adduct is obtained. The formation of the new C–C and C–O bonds occurs through a concerted but slightly asynchronous approach.

**Scheme 45:**
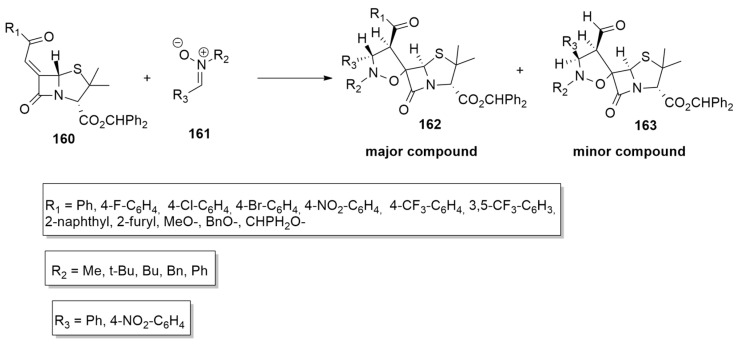
Synthesis of chiral spiro-b-lactams from 6-alkylidenepenicillanates.

The 1,3-dipolar cycloaddition of 6-alkylidenepenicillanates **160**, in toluene at 80 °C for 24 h, with nitrones **161** was employed by Alves et al. to synthesize different chiral spiroisoxazolidine-b-lactams **162** (major) and **163** (minor) (Scheme 45) [65].

The reaction proved to be stereo- and regioselective, giving rise to **162** as the main adduct with a yield varying between 26 and 70%.

A complete conformational study of the spiroisoxazolidine-β-lactams **162** and **163** was carried out at the B3LYP/6-31G(d) level. The *endo* compound **162** was shown to be more stable than the *exo* one **163** with a butterfly-like geometry characterized by an open shape conformation.

**Scheme 46:**
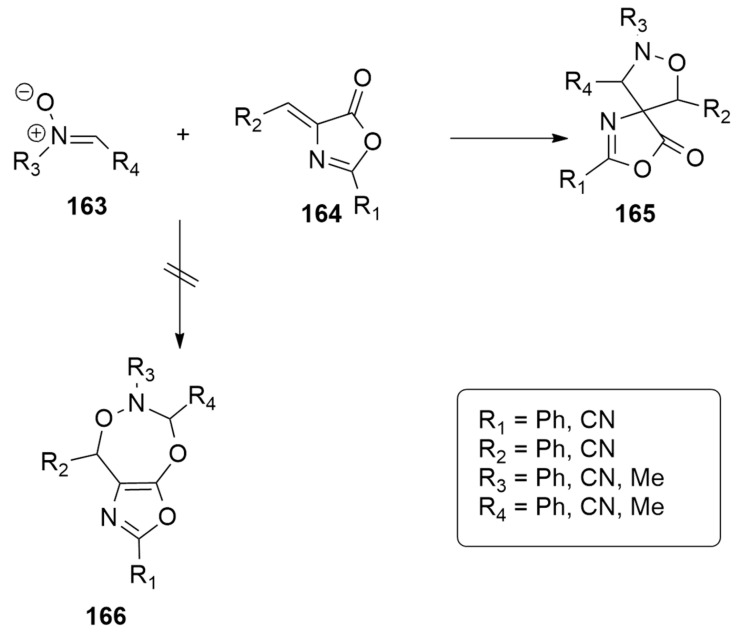
Synthesis of compound **166**.

Other DFT studies were performed at the M062X/6-311G(d,p) level on the cycloaddition reaction of C,N-disubstituted nitrones **163** with disubstituted 4-methylene-1,3-oxazol-5(4H)-one **164**. The authors highlight that the dipole, contrary to the mechanism reported by Mekheimer et al. [66], adds chemo selectively to the olefinic bond, forming the corresponding spiro cycloadduct **165**, rather than to the methylene carbon and the carbonyl oxygen in a [4 + 3] cycloaddition, leading to **166**. Furthermore, it was found that the presence of electron-withdrawing groups in the dipole reduces the energy barriers, while it is increased in the presence of electron-donating groups [67] (Scheme 46).

The lack of regioselectivity in 1,3-DC of methylenecyclopropane **170**, compared to the high regioselectivity of other 1,1-disubstituted alkenes, has been computationally investigated through DFT calculations, to rationalize the experimental finding [68].

**Scheme 47:**
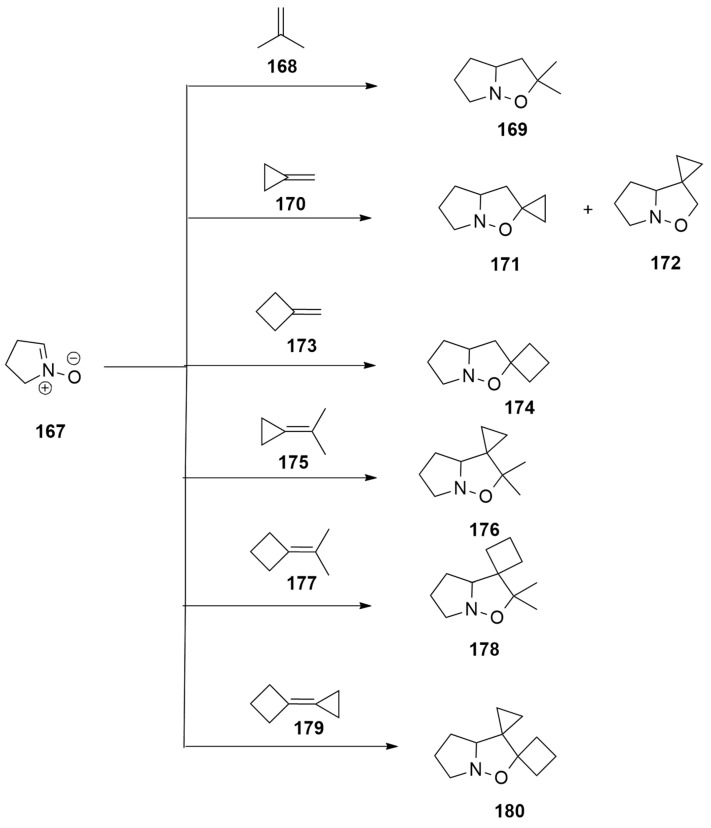
Computed 1,3-dipolar cycloadditions involving different 1,1-disubstituted alkenes.

Considering the cycloaddition involving nitrone **167** and isobutene **168** (Scheme 47), only the 5,5-dimethyl-substituted isoxazolidine **169** is experimentally obtained with the two methyls on position 5. The TS leading to this cycloadduct is lower in energy with respect to that leading to the other possible regioisomer (the 4,4-dimethyl-substituted isoxazolidine). The calculated kinetic ratio is rationalized through the charge distribution and the orientation of the dipole moment of alkene.

The reaction with methylenecyclopropane **170**, experimentally determined, leads to the formation of a regioisomeric mixture of **171** and **172** in a 65:35 ratio, respectively, with the 5-spiro-fused isoxazolidine **171** as the major product. Instead, the reaction carried out with **173** provides only the methylenecyclobutane **174**. The calculated barriers gave explanation to these results. For **173**, μ is oriented toward the cyclopropane. The two alkenes (**170** and **173**) present a different reactivity determined by electrostatic and kinetic aspects. In the reaction involving **170**, a lower EDG effect of the substituents is detected, and this results in a lack of the regioselectivity. Whereas, in the case of **173**, the charge distribution of the alkene leads to furnishing the 5-spiro-fused product **174**.

The study was also extended to isopropylidenecyclopropane **175** and isopropylidenecyclobutane **177.** The 1,3-dipolar cycloaddition between **175** and nitrone **167** led only to the 5,5-dimethylisoxazolidine **176**, in which the barrier of the other orientation was the highest. Instead, the difference of the regioisomeric barriers of the reaction between **177** and nitrone is much lower with 5,5-dimethylisoxazolidine **178**, which is slightly kinetically favored. Finally, the reaction of nitrones with cyclobutylidenecyclopropane **179** was studied. Experimentally, only cycloadduct **180** bearing cyclopropane on C4 was obtained. Calculations showed that this product is both kinetically and thermodynamically favored.

**Scheme 48:**
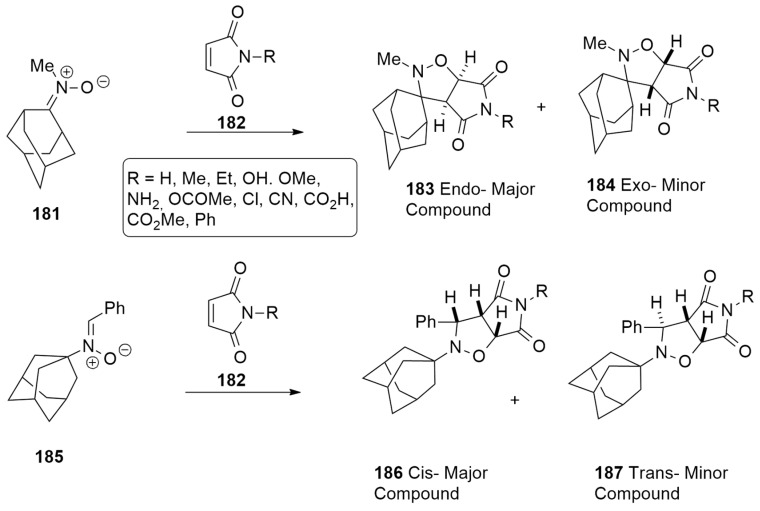
Synthesis of isoxazolidines through 1,3-DC of adamantine aldo and ketonitrones.

In 2020, the 1,3-DC of adamantine aldo and ketonitrones with maleimides leading to isoxazolidines appeared in the literature [69]. From an experimental point of view, the reaction was conducted in toluene at 110 °C. In the case of **185**, a diastereomeric mixture was obtained, while in the case of **181**, only one isomer is achieved (Scheme 48). However, the authors did not specify whether this isomer was the *exo* or *endo* one and if its formation was due to thermodynamic or kinetic factors. 

**Figure 24:**
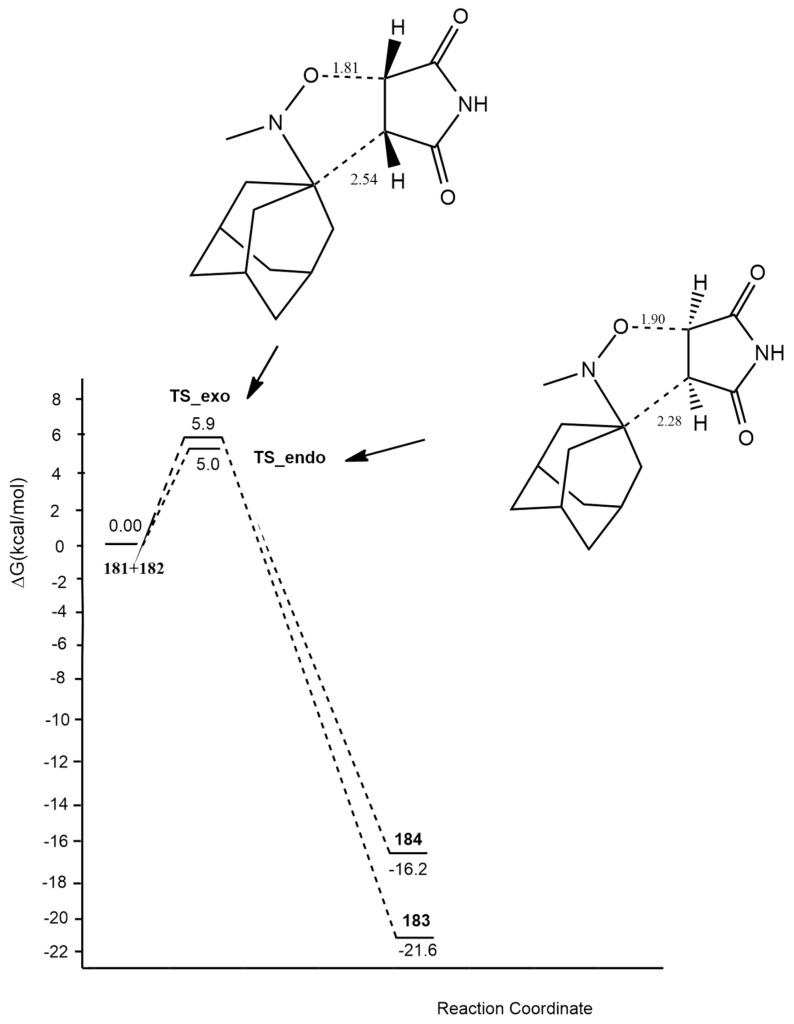
Free energy profile of reaction of 181 with maleimide in experimental conditions (T = 110 °C).

Considering the importance of adamantane and isoxazolidine moieties in the field of medicinal chemistry, the stereo selective reaction of keto- **181** and aldonitrone **185** containing an adamantine moiety with maleimides **182** was computationally investigated by Umar et al. [70] using DFT methods at the M06-2X/6-311++G(d,p) level and the polarizable continuum solvent (PCM) model. Both reactions proceed in a concerted, pericyclic manner. In detail, the reaction of **181** with maleimide **182** proceeds through two TSs, leading to the *endo*- or *exo*-stereoisomer with activation barrier values of 2.8 and 4.5 kcal/mol at room temperature, respectively (Figure 24).

Calculations were repeated at experimental conditions and in particular considering T = 110 °C. The *endo* adduct is thermodynamically preferred, while the two TSs are very close in energy. Since only an isomer was experimentally detected, the less stable *exo* one is probably converted into the *endo* one. 

**Figure 25:**
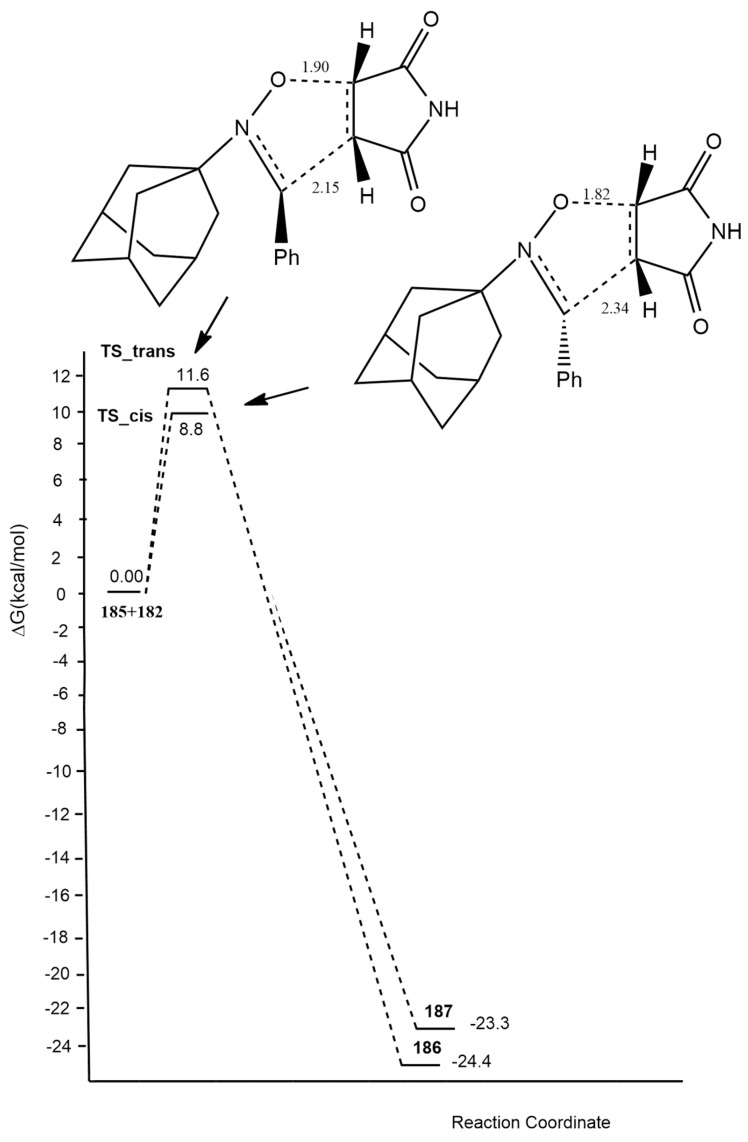
Free energy profile of reaction of **185** with maleimide in experimental conditions (T = 110 °C).

The reaction of aldonitrone **185** with maleimide leads to *trans* and *cis*-stereoisomers with activation barriers of 10.3 and 8.0 kcal/mol, respectively (Figure 25). The reaction is exergonic, and from the energetic point of view, the stereoisomers are thermodynamically similar and kinetically different. The reaction is under kinetic control, and the *cis* adduct is preferentially obtained. 

Table 2 reports and summarizes the key findings of the reviewed studies involving 1,3-dipoles of allyl type.

## 3. Conclusions

The classical 1,3-dipolar cycloaddition (1,3-DC) is considered one of the most exploited strategies for the synthesis of five-membered heterocycles, allowing the introduction of several stereogenic centers in a stereospecific manner and in a single step. The mechanistic aspects of this reaction and in particular the regio-, diastereo- and enantioselectivity are rationalized and predicted using quantum mechanical calculations. The DFT calculations have proven to be particularly effective to explain the outcome of the cycloaddition and predict the products obtained during the reaction. 

In conclusion, in this review, we have highlighted the importance of DFT calculations, focusing our observation on the mechanistic insights on aza-ylides, nitrile oxides, azomethine ylides and nitrones as dipoles which have appeared in the literature in the last 5 years. Moreover, a careful analysis of the different computational approaches allows us to conclude that although several new functionals have been introduced, such as the Thrular’s hybrid metafunctionals, to date, B3LYP remains the most used for the study of this type of organic reaction by the scientific community. However, the growing interest toward new catalysts, in particular enzymatic but also metallic, opens the way to the testing of new future combinations of computational methods, which are able to predict the selectivity of 1,3-DC reactions.

## Figures and Tables

**Table 1:** Summary of the key findings of the reviewed studies involving 1,3-dipoles of propargyl-allenyl type (reagents, reaction conditions, catalysts, and computational methods).

1,3-Dipoles of Propargyl-Allenyl Type			
Aza-Ylides			
Reagents	Reaction Conditions	Catalyst	Level of Calculations
methylazide + propine	Δ, catalyst	Cu (I)	B3LYP/6-31G(d) and LANL2DZ for Cu
methylazide + 2-butine or cycloalkyne	Δ	/	M062X/6-311++G(d)
methyl azide and various allenes	Bu_4_NF/CsF, CH_3_CN	/	PB86/TZ2P
methyl azide and propyne	r.t., catalyst	Ag(I)	B3LYP/6-31G(d) and LANL2DZ for Ag
differently substituted azides + alkynes, cyanoalkynes, thioalkynes, and ynamides	Cp_2_Ni (10 mol %), Xantphos (10 mol %), Cs_2_CO_3_ (1 eqv), DCM, r.t., 24 h	Ni	M06/6-31G(d,p) and cc-pVTZ for Ni
lithiated trimethylsilyldiazomethane and α-azido ketones	LTMSD, −78 °C, 1h then r.t.	/	M06/6-31+G
arylazide + phenylacetylene	CuSO_4_·5H_2_O/5 Na^+^ L-Asc. 1 eq, DMF (1mL), t = 42 min, 25 °C, air	Cu	M06/6-311+G(d,p) SDD
benzylazide+terminal alkynes	50 °C, catalyst, EtOH	Cu(I), Au_4_Cu_4_/CNT	PBE/ECP6MWB and def2-TZVPP for Cu, def2-SVP for other
1,2-diboraallene+2,6-diisopropylphenyl azide	Toluene/n-hexane/r.t.	/	B3LYP/6-311G(d,p)
alkyne+(azidomethyl)benzene	(a) Cs_2_CO_3_, DMF, r.t.; (b) THF/TEA (1:1), 55–60 °C, CuBr(PPh_3_)_3_, 5.5 h,	Cu(I)	B3LYP/6-311++G(d,p) level and B3LYP/LANL2DZ for metals
2-butylidenemalononitrile + phenylazide	DBU, DMSO-d6 at 50 °C	/	M06-2X/6-31+G(d,p)
azide + alkyne	15 mol % catalyst, 45 °C, 30 min and water	CuC_20_H_20_N_2_)PPh_3_Cl and Cu(C_20_H_20_N_2_)PPh_3_Br	LDA/PWC
1,2,3,4-tetrazole + phenylacetylene	Mn(TPP)Cl, Zn, C6H6, 100 °C, 24 h	manganese-copper/zinc	M06/6-311++G** for all other atoms and LANL2DZ for metals
phenylacetylene + phenyl azide	carbon nanotubes	/	QM/MM ONIOM B97D/UFFs
**Nitrile Oxides**			
**Reagents**	**Reaction conditions**	**Catalyst**	**Level of calculations**
N-tert-Butoxycarbonyl [1-Chloro-1-(hydroxyimino)-3-phenylpro-pan-2-yl)]carbamate + N-benzyl-3-pyrroline	Base/r.t.	/	CPCMHCTC/6-11+G(d,p)//HCTH/6-31+G(d)
nitrile oxide + graphene sheets	MW	/	(U)M06-2X/6-31+G(d,p)
acetonitrile oxide +(*1S*,*2R*,*4S*)-2-cyano-7-oxabicyclo[2.2.1]hept-5-en-2-yl acetate derivatives	r.t.	/	ωB97X-D/6-311G(d)
oxime + t-but acrylate	Et_4_NCl/CH_3_CN	/	M06-2X/Def2TZVP
nitrile oxides + cyclodienes	MeCN, 25 °C or 70 °C	/	M06-2X/6-311+G(d,p)

**Table 2:** Summary of the key findings of the reviewed studies of azomethyne ylides and nitrones (reagents, reaction conditions, catalysts, and level of calculations).

1,3-Dipoles of Allyl Type			
Azomethyne Ylides			
Reagents	Reaction Conditions	Catalyst	Level of Calculations
ortho hydroxy imine + nitro alkanes	p-xylene, r.t., cat	N,N′−bis[3,5−bis(trifluoromethyl)phenyl]−thiourea	M06-2X/6-31g(d,p)
allyl ammine + aldehydes	PhMe, r.t/ maleimide, 150 °C, PhMe	/	B3LYP/def2SVP
azomethine ylide+ acrylonitriles		Cu(CH_3_CN)_4_BF_4_ and chiral phosphine−urea bifunctional ligand	M11/6–311+G(d,p)
glycine imino ester + beta-fluoroalkyl alkenyl arylsulfones	THF, Et_3_N	BINAP-Cu(II)	M06-L/def2-TZVPP
ninhydrin + L-Prolin	MeOH, r.t.	/	M11 / cc-pVDZ
imino esters + electron deficient alkenes	Et_3_N, PhMe	L1-CuOTf·PhMe or L2-AgSbF_6_	B3LYP/6-31G(d)
N-ethylglycine (50equiv) + paraformaldehyde	o-ClC_6_H_4_, 120 °C	Gd_3_N@Ih-C80	BP86-D2/TZP
azomethine ylide+[60]fullerene	PhMe, Δ, Ar	/	B3LYP/6-31+G(d,p)/C-PCM = toluene//OLYP/6-31G(d):PM6
azomethine ylide +nitrostyrene	CTPRs, THF, r.t.	/	ONIOM M06-2X/6-311+G(2d,2p):dreiding//B3LYP/6-31G-(d,p):dreiding
azomethine ylide + methyl cinnamate or N-enoyl oxazolidinone	[Ir], TMDS, PhMe, r.t.	iridium complex	BP86/TZ2P
azomethine ylide + alkenyl derivatives	THF, r.t.	Cu(I)	M06-L/6-31G(d), SDD for Cu
azomethine ylide + cyclopropenesor maleimide	1,4-dioxane, 100 °C, N_2_	/	M11/cc-pVDZ
amino acids + isatins	EtOH, 50 °C	Ni(II)	PBE0-D3BJ/def2SVP
azomethine ylide + arylidenes or phenylmaleimide	AcONa, PhMe, r.t. or 110 °C	/	wB97xd/6-31G(d,p)
glycine imino ester + *N*-phenylmaleimide	AcONa, TEA, EtOH, reflux	AgOAc	wB97xd/6-31G(d,p)/ Lanl2dz for Ag
**Nitrones**			
nitrones + oxa(aza)bicyclic alkenes	PhMe, 60 °C	/	B3LYP/6-311G(d,p)
N-methyl-C-ethoxycarbonylnitrone + allyl-heptaisobutyl-POSS	100W, PhMe, 110 °C	/	B3LYP/6-31G(d)
nitrone +6-alkylidenepenicillanates	PhMe, 80 °C	/	B3LYP/6-31G(d) level
C,N-disubstituted nitrones + disubstituted 4-methylene-1,3-oxazol-5(4H)-one	PhMe, reflux	/	M062X/6-311G(d,p)
adamantine aldo and ketonitrones + maleimides	PhMe, reflux	/	M06-2X/6-311++G(d,p)
